# Shell Disease Syndrome Is Associated with Reduced and Shifted Epibacterial Diversity on the Carapace of the Crustacean Cancer pagurus

**DOI:** 10.1128/spectrum.03419-22

**Published:** 2022-11-07

**Authors:** Nils Bergen, Philipp Krämer, Julia Romberg, Antje Wichels, Gabriele Gerlach, Thorsten Brinkhoff

**Affiliations:** a Institute for Chemistry and Biology of the Marine Environment, University of Oldenburg, Oldenburg, Germany; b Institute for Biology and Environmental Science, University of Oldenburg, Oldenburg, Germany; c Alfred-Wegener-Institute, Helmholtz Centre for Polar and Marine Research, Biologische Anstalt Helgoland, Helgoland, Germany; d Helmholtz Institute for Functional Marine Biodiversity (HIFMB), Oldenburg, Germany; University of Prince Edward Island

**Keywords:** *Crustacea*, microbiome composition, shell disease, *Aquimarina*, *Rhodobacteraceae*, microbe-host interaction

## Abstract

Cancer pagurus is highly susceptible to shell disease syndrome. However, little is known about concomitant changes in the epibacterial community. We compared the bacterial communities of black spot affected and nonaffected areas of the carapace by amplicon sequencing of 16S rRNA genes and 16S rRNA. Within each spot, bacterial communities of affected areas were less diverse compared to communities from nonaffected areas. Communities of different affected spots were, however, more divergent from each other, compared to those of different nonaffected areas. This indicates a reduced and shifted microbial community composition caused by the black spot disease. Different communities found in black spots likely indicate different stages of the disease. In affected areas, *Flavobacteriaceae* rose to one of the most abundant and active families due to the increase of *Aquimarina* spp., suggesting a significant role in shell disease syndrome. We isolated 75 bacterial strains from diseased and healthy areas, which are primarily affiliated with *Proteobacteria* and *Bacteroidetes*, reflecting the dominant phyla detected by amplicon sequencing. The ability to degrade chitin was mainly found for *Gammaproteobacteria* and *Aquimarina* spp. within the *Flavobacteriia*, while the ability to use *N-*acetylglucosamine, the monomer of the polysaccharide chitin, was observed for most isolates, including many *Alphaproteobacteria*. One-third of the isolates, including most *Aquimarina* spp., showed antagonistic properties, indicating a high potential for interactions between the bacterial populations. The combination of bacterial community analysis and the physiological properties of the isolates provided insights into a functional complex epibacterial community on the carapace of C. pagurus.

**IMPORTANCE** In recent years, shell disease syndrome has been detected for several ecologically and economically important crustacean species. Large proportions of populations are affected, e.g., >60% of the widely distributed species Cancer pagurus in different North Sea areas. Bacteria play a significant role in the development of different forms of shell disease, all characterized by microbial chitinolytic degradation of the outer shell. By comparing the bacterial communities of healthy and diseased areas of the shell of C. pagurus, we demonstrated that the disease causes a reduced bacterial diversity within affected areas, a phenomenon co-occurring also with many other diseases. Furthermore, the community composition dramatically changed with some taxa rising to high relative abundances and showing increased activity, indicating strong participation in shell disease. Characterization of bacterial isolates obtained from affected and nonaffected spots provided deeper insights into their physiological properties and thus the possible role within the microbiome.

## INTRODUCTION

Cancer pagurus, commonly known as the edible or brown crab, and other crustacean species, including shrimps and lobsters, are nowadays recognized to be strongly affected by shell diseases, both in natural and captive populations. Shell diseases might be caused by different factors, like pollutants, mechanical damage, and temperature change, but are mainly promoted by microbial processes, e.g., chitin degradation in the cuticle (1–3). C. pagurus has an important ecological role as a predator, especially of mollusks, in the North Sea and the northeast Atlantic (4) and is commercially the most important crab species in Western Europe with catch rates up to 45,000 tons per year (5, 6). In previous studies, the prevalence of shell disease in populations has been recorded for large percentages (>50%) of C. pagurus populations (1, 7). Black spot shell diseases are named variously, such as black necrotic disease, burned spot disease, or rust disease, and are characterized by necrotic lesions of the crustacean carapaces. Thus, the black spot shell syndrome is responsible for the economic losses of the crab fisheries in terms of marketability (8). Here, we focused on the black spot syndrome, which is characterized by black necrotic lesions of the exoskeleton that, in advanced stages (9), are accompanied by pitting on the inner surface of the exoskeleton and can eventually lead to animal death (10).

In previous studies, injection of extracellular products of Pseudoalteromononas atlantica, isolated from shell disease-infected C. pagurus, into healthy crabs caused rapid death (11), indicating high relevance of the bacterial extracellular products in the late phases of shell disease. Contrary to other microbial diseases of crustaceans, which are caused by single pathogens (12), the causative agents of the black spot syndrome are versatile. Vogan et al. (1) emphasized the complexity of shell diseases and considered the role of microbial interactions in the development of black spots. The causes of the black spot shell syndrome have intensively been studied in the American lobster, Homarus americanus (e.g., Bell et al. (13), Castro and Angell (14), Castro et al. (15), and Meres et al. (16)), and there is a consensus that such diseases arise from a combination of multiple biotic and abiotic stressors that increase the crustacean’s susceptibility to potentially pathogenic bacteria (15).

So far, only a few studies investigated the microbiota of C. pagurus with a focus on potential pathogenic bacteria contributing to the black spot shell syndrome. Recently, we provided an overview of the epibiotic bacterial community associated with the shell of C. pagurus in different environments (17). We found that the bacterial community on C. pagurus was similar to those of other marine crustaceans, especially the American lobster. Furthermore, we identified many taxa with potential chitinolytic activity, e.g., representatives of the genera *Aquimarina*, *Kiloniella*, *Thalassobius*, and *Leucothrix* (17). These genera were also found in shell disease-affected lesions of H. americanus specimens (16). *Aquimarina* sp. I32.4 has been described in several studies as a potential causative agent in the bacterial community of H. americanus (16, 18, 19). A comparison of the total bacterial communities of healthy and affected areas of the carapace of C. pagurus has not been performed yet. However, results from detailed studies with different crustacean species living in different environments are needed to identify the bacteria that are important for black spot disease in general.

In this study, we analyzed the diversity, relative abundance, and activity of epibacterial communities living in healthy and black spot-affected areas of the carapace of several C. pagurus specimens to elucidate changes in bacterial communities occurring during the disease and identify key populations at low taxonomic levels. In addition, we isolated 75 different bacterial strains from healthy and black spot-affected areas and investigated their physiological characteristics, i.e., chitinolytic activity and antagonistic properties, both important for interactions within the bacterial community and with their host.

## RESULTS

### Bacterial diversity on the carapace of C. pagurus.

**FIG 1:**
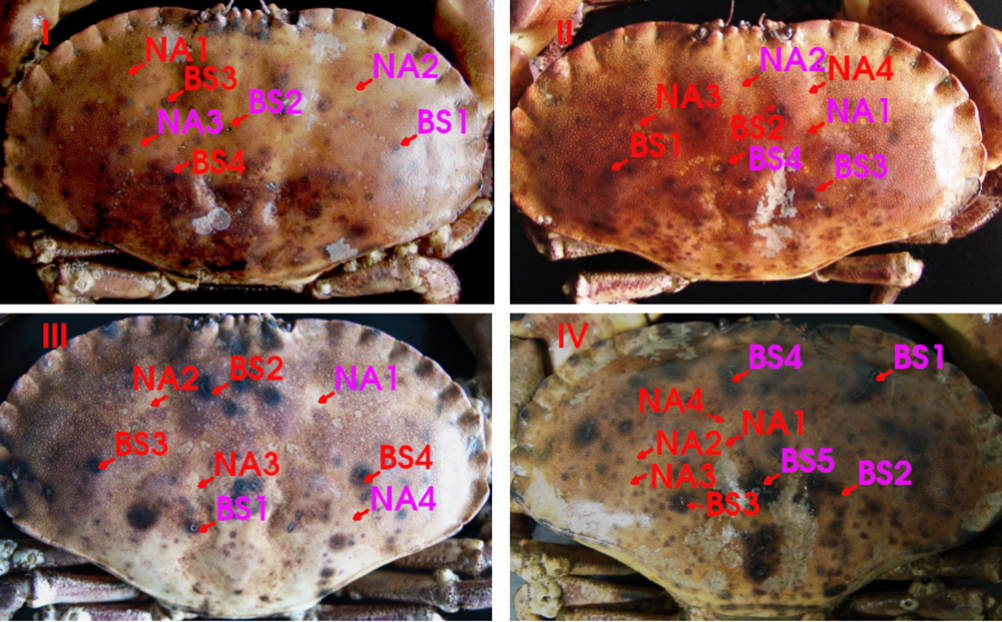
Sampled nonaffected (NA) and black spot (BS) affected areas of the carapaces of the Cancer pagurus specimens investigated in this study. Red arrows indicate sampling spots. Purple spots indicate areas where isolates had also been taken.

**FIG 2:**
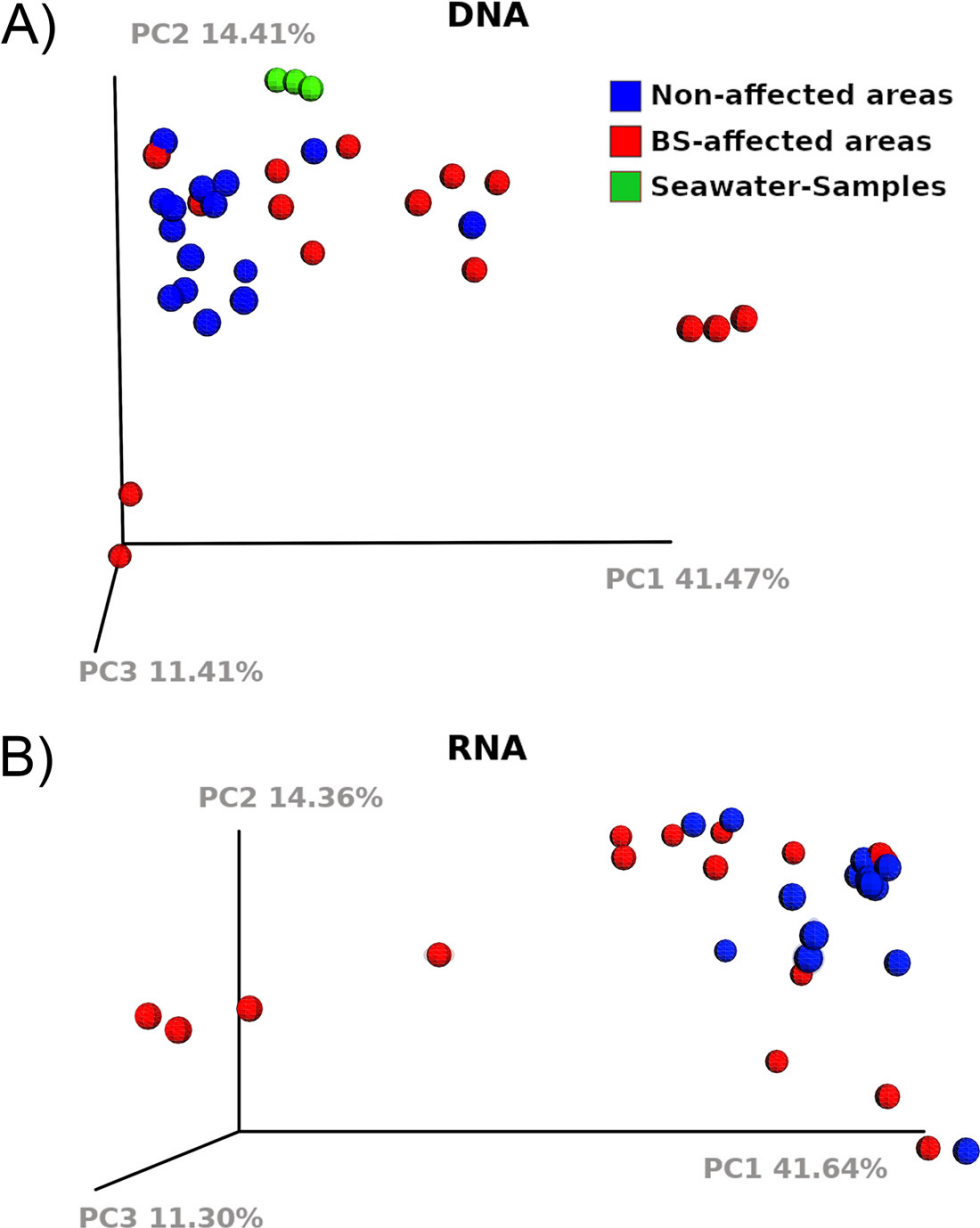
Principle coordinate analysis of all samples with more than 2000 sequences, separately plotted for (A) DNA samples (30 + 3 seawater samples) and (B) RNA samples (30). All samples were randomly jackknifed to 2000 sequences per pool and the first three principal coordinates are plotted (PC1, PC2, and PC3).

The bacterial communities of 15 samples from nonaffected (NA) and 17 from black spot syndrome affected (BS) spots of the carapace of four intermoult males of C. pagurus (Fig. 1) were analyzed by amplicon sequencing of extracted DNA and RNA. Specimens of C. pagurus selected for the analysis were of similar size and averagely affected by shell disease syndrome (Fig. 1), i.e., specimens with fully healthy carapace and with heavy shell disease were not considered for sampling. Samples taken from BS and NA spots generally differed from one another, as indicated by principal coordinate analysis (PCoA; Fig. 2A). In the PCoA, the NA samples produced a cluster more distinct in diversity compared to the cluster of the BS samples. However, it should be noted that there was some overlap of samples from the BS areas with the cluster of samples from NA areas. Compared to the carapace samples, the seawater samples were relatively even and formed a distinct cluster in the PCoA analysis (Fig. 2A). In general, NA communities were found to be more diverse than BS communities in the same individual of C. pagurus (similarly for DNA and RNA pools; Table 1). The number of operational taxonomic units (OTUs) found in NA areas was higher than in BS areas (between 79 and 167 more OTUs in NA areas on the DNA level, and 122 to 193 OTUs more on the RNA level, Table 1). Diversity indices calculated with Shannon-Wiener and Simpson evenness generally resulted in a significantly higher evenness and diversity of NA (Table 1; the number of rarified OTUs, N = 4, *P* < 0.05; Shannon-Wiener, N = 4, *P* < 0.05; Simpson evenness, N = 4, *P* < 0.05). In contrast to the lower diversity of BS compared to NA ranges in each C. pagurus individual (Table 1), this did not result in a higher similarity of BS samples compared to NA ranges across all individuals (Fig. 2A and B).

**TABLE 1:** Alpha diversity calculated from 2000 rarefied reads for each pool of sequences. Four different specimens (Spec) samples were taken on different sites of the carapace (N) from healthy (NA) and shell diseased (BS) individuals^*a*^

Specimen	Group	N	OTUs in rarefied reads^*b*^		Shannon-Wiener^*b*^		Simpson evenness^*b*^	
			Mean	Diff.	Mean	Diff.	Mean	Diff.
DNA - rarefied to 2000								
1	NA	3	289 ± 76	−153	6.55 ± 0.36	−1.68	0.13 ± 0.05	−0.01
	BS	4	136 ± 47		4.87 ± 1.36		0.12 ± 0.06	
2	NA	4	298 ± 157	−167	6.35 ± 2.13	−2.19	0.19 ± 0.11	−0.08
	BS	4	131 ± 154		4.16 ± 2.47		0.11 ± 0.08	
3	NA	4	341 ± 68	−99	7.27 ± 0.31	−2.76	0.23 ± 0.01	−0.12
	BS	3	242 ± 306		4.51 ± 3.29		0.11 ± 0.10	
4	NA	4	268 ± 31	−79	6.71 ± 0.16	−2.00	0.18 ± 0.04	−0.10
	BS	5	189 ± 104		4.71 ± 1.85		0.08 ± 0.04	
RNA - rarefied to 2000								
1	NA	3	344 ± 15	−193	7.13 ± 0.16	−2.33	0.21 ± 0.05	−0.10
	BS	4	151 ± 57		4.80 ± 1.17		0.11 ± 0.06	
2	NA	4	341 ± 166	−122	6.32 ± 2.35	−1.14	0.16 ± 0.10	−0.00
	BS	4	219 ± 216		5.18 ± 3.13		0.16 ± 0.10	
3	NA	4	388 ± 118	−163	7.28 ± 0.64	−2.49	0.18 ± 0.06	−0.06
	BS	3	225 ± 233		4.79 ± 2.70		0.12 ± 0.11	
4	NA	4	326 ± 33	−125	7.03 ± 0.19	−1.96	0.20 ± 0.02	−0.11
	BS	5	201 ± 113		5.07 ± 1.75		0.09 ± 0.04	

a From each community pooled DNA and RNA were sequenced. Based on the rarefied pools' mean number of OTUs,mean Shannon-Wiener and mean evenness by 1/Simpson corrected as average over N carapace sites on one individual is calculated. For each metric means, ± s.d. and difference in means for NA and BS samples per individual (Diff.) are calculated. Paired differences were tested with theWilcoxon rank sum test for each metric over individuals with W = 0 and significance.

b *P* < 0.05.

### Composition of bacterial communities in NA and BS areas.

**FIG 3:**
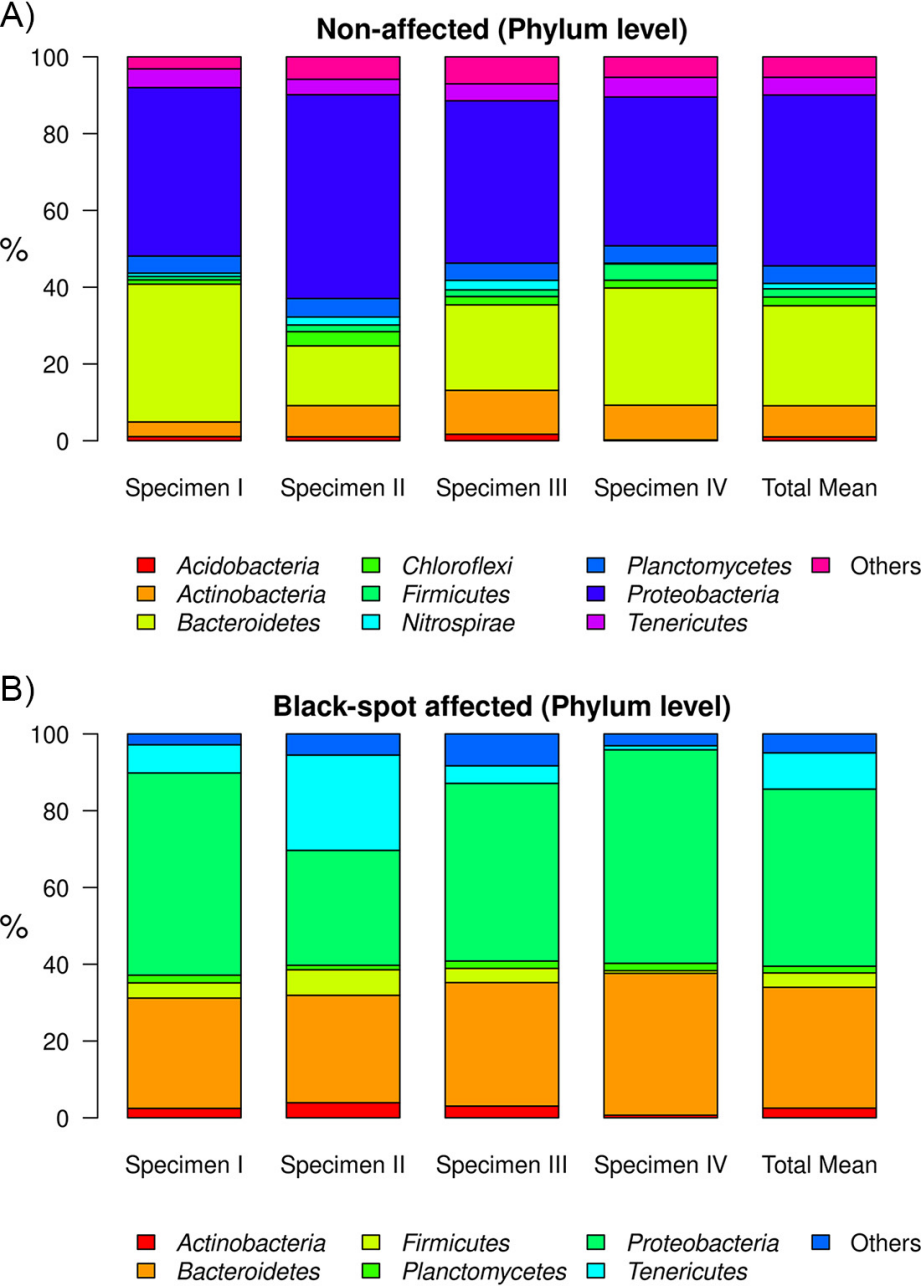
Relative abundance (and mean) of bacterial phyla present in the epibacterial biofilms of four Cancer pagurus specimens (average value for each specimen derived from 65 samples). Samples were taken from nonaffected (A) and black spot syndrome (B) affected spots.

In the NA areas of the carapace of C. pagurus, the most abundant phyla (≥1%) of the epibiotic bacterial communities, based on DNA data, were *Proteobacteria* (44.5 ± 6.1%), *Bacteroidetes* (26.1 ± 9.0%), *Actinobacteria* (8.0 ± 3.3%), *Planctomycetes* (4.5 ± 0.1%), *Tenericutes* (4.5 ± 0.5%), *Chloroflexi* (2.2 ± 1.1%), *Firmicutes* (2.0 ± 1.5%), *Nitrospirae* (1.3 ± 1.1%), and *Acidobacteria* (1 ± 0.6%). On average, these nine phyla accounted for 94.7% of the microbial community (Fig. 3). In the BS areas only six of these nine phyla were present ≥1% (based on DNA data), i.e., *Proteobacteria* (46.1 ± 11.5%), *Bacteroidetes* (31.5 ± 4.1%), *Tenericutes* (9.4 ± 10.6%), *Firmicutes* (3.6 ± 2.6%), *Actinobacteria* (2.4 ± 1.5%), and *Planctomycetes* (1.6 ± 0.4%), comprising overall 94.6% of the bacterial community (Fig. 3). The one-third decrease in common phyla already indicates lower diversity at this high taxonomic level.

At the family level, based on DNA data, most amplicon sequences from NA with ≥1% abundance were classified as *Flavobacteriaceae* (11.6 ± 8.7%), *Saprospiraceae* (11.4 ± 7.6%), *Hyphomonadaceae* (5.8 ± 2.8%), *Arenicellaceae* (5.0 ± 7.0%), *Mycoplasmataceae* (4.5 ± 0.5%) or *Rhodobacteraceae* (4.7 ± 2.5%). In BS areas most abundant were *Flavobacteriaceae* (25.2 ± 4.2%), *Arenicellaceae* (8.2 ± 6.9%), *Rhodobacteraceae* (6.4 ± 1.5%), and *Mycoplasmataceae* (9.3 ± 10.6%). Compared to NA areas, *Flavobacteriaceae* (25.2 ± 4.2%) were strongly increased in BS areas, while *Saprospiraceae* (3.9 ± 0.9%) and *Hyphomonadaceae* (3.4 ± 2.2%) strongly decreased. Some families were absent from the community in BS samples compared to NA in DNA samples, such as the Sva0096 marine group and some uncultured members within the *Acidimicrobiales*, *Granulosicoccaceae*, or *Planctomycetaceae*. A shift in the epibacterial diversity on the carapace of C. pagurus was additionally visible by the decreased number of families contributing to the community for OTUs with >1% abundance in BS samples (12 families) compared to NA samples (17 families) (see Supplemental File 2).

Based on DNA analyses, we found an average of 22.9 ± 2 different genera with an abundance of ≥1% in individual communities in NA areas, compared with only 17 ± 1 for BS samples (Table 2). The number of different genera identified per specimen ranged between 21 and 26 in NA areas. For BS areas, the number of different genera identified per specimen was between 15.8 and 18.5. Per individual, different patches of BS showed a significantly higher variation in the number of different genera than NA spots (Table 2). This higher variation in BS samples resulted from an overall higher number of genera found among BS samples (105) than in NA samples (75). Based on all genera (N = 859) with at least 1% abundance in at least one sample, only 23 genera were found unique for NA samples compared to 53 genera unique for BS samples (Supplemental File 2). This higher variation was also obvious from the PCoA, showing a lesser spread of NA samples compared to BS samples for both RNA and DNA samples (Fig. 2). At first glance, this seemed contradictory, but it demonstrated the specificity of the bacterial communities and emphasized the importance of analyzing the data based on the pairwise comparison of the BS and NA plots for each individual.

**TABLE 2:** Number of genera contributing with at least 1% to individual bacterial communities, based on DNA amplicon sequencing data in samples from nonaffected (NA) and black spot syndrome affected areas (BS) of the four C. pagurus specimens

Specimen	Sample	NA		Sample	BS	
		No. of genera	Mean		No. of genera	Mean
1	I-NA1	23	22 ± 1	I-BS1	26	18.5 ± 7
	I-NA2	22		I-BS2	20	
	I-NA3	21		I-BS3	9	
				I-BS4	19	
2	II-NA1	26	22.5 ± 6	II-BS1	26	17.3 ± 11
	II-NA2	13		II-BS2	26	
	II-NA3	25		II-BS3	4	
	II-NA4	26		II-BS4	13	
3	III-NA1	27	26 ± 2	III-BS1	8	16.5 ± 12
	III-NA2	23		III-BS2	5	
	III-NA3	28		III-BS3	29	
	III-NA4	26		III-BS4	24	
4	IV-NA1	18	21 ± 3	IV-BS1	20	15.8 ± 6
	IV-NA2	24		IV-BS2	21	
	IV-NA3	18		IV-BS3	13	
	IV-NA4	24		IV-BS4	6	
				IV-BS5	19	
Total mean	NA		22.9 ± 2	BS		17 ± 1

### Activity levels in NA and BS areas.

**FIG 4:**
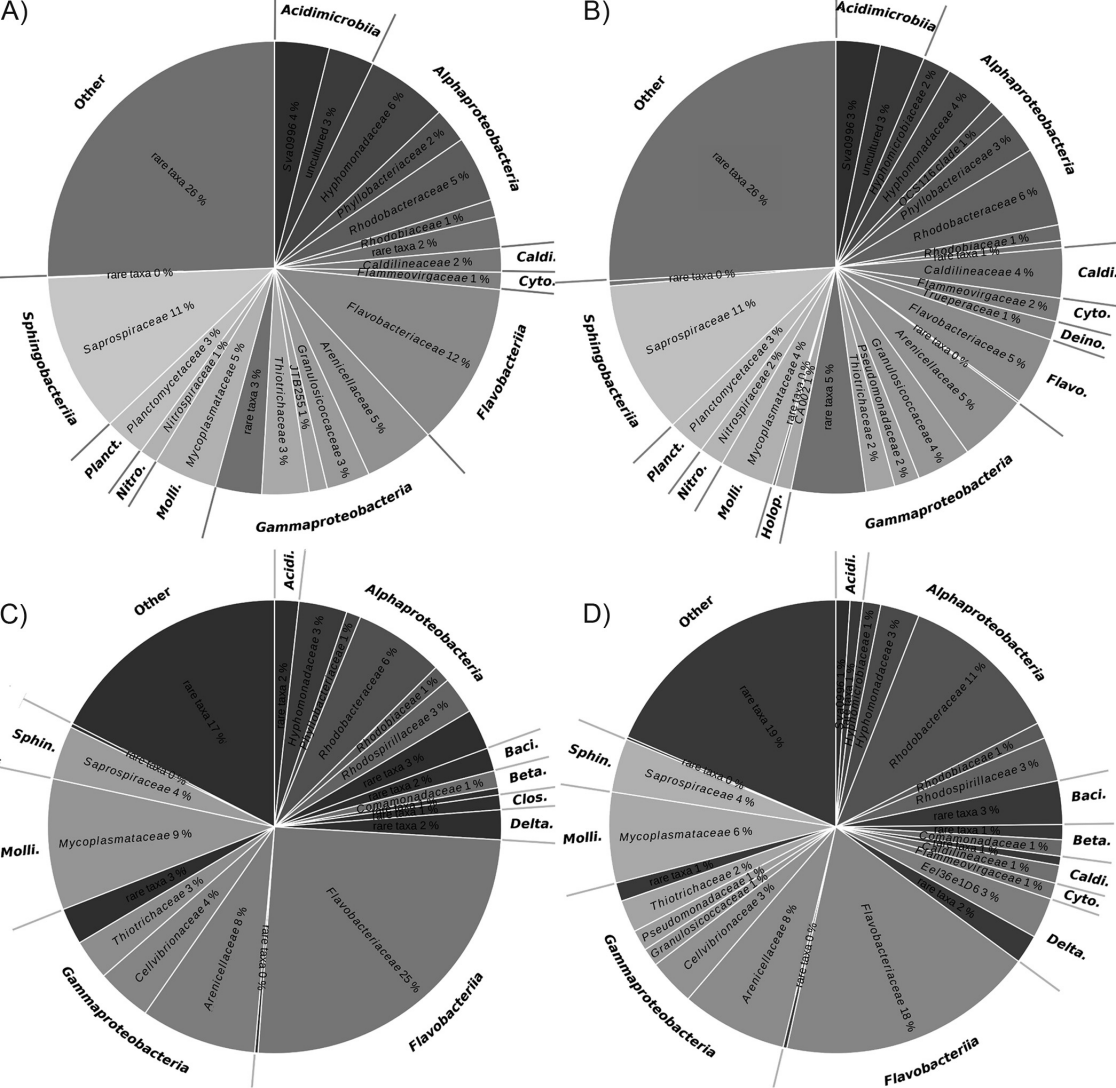
Relative abundance of taxa on the level of the family; based on amplicons of 16S rRNA gene sequences, including all OTUs with abundance ≥ 1% averaged for all samples of (A) NA-DNA, (B) NA-RNA, (C) BS-DNA, and (D) BS-RNA. Families are grouped according to their phylogeny in classes. Classes are abbreviated as follows: *Baci*, *Bacilli*; *Beta*, *Betaproteobacteria*; *Caldi*, *Caldilineaceae*; *Cyto, Cytophagia*; *Clos, Clostridia*; *Deino, Deinococci*; *Delta*, Deltaproteobacteria; *Flavo*, *Flavobacteria*; *Holop*, *Holophagae*; *Molli*, *Mollicutes*; *Nitro*, *Nitrospira*; *Plancto*, *Planctomycetaceae; Sphin*, *Sphingobacteriia*. Families with less than 1% abundance were summed up as rare taxa within the classes. The category “Other” included all families below the threshold of 1% abundance not belonging to a class with at least one family with more than 1% abundance.

Due to strong differences in the activity of single taxa within higher taxonomic levels, summarizing analyses for phylum, class, and order level was not performed. At the family level, the highest activity in NA areas was observed for the *Saprospiraceae* (11%), identical to their relative abundance based on DNA (Fig. 4). In NA areas relative abundance and activity were similar for almost all abundant families except the *Flavobacteriacea*e, in which activity was only 5% of the total community compared to 11.6% abundance on a DNA level. In BS areas, the activity of the *Flavobacteriaceae* increased to 18%, making them the family with the overall highest activity, but the activity level was still lower than their relative abundance based on DNA (25.2%). Based on RNA analysis the *Flavobacteriaceae* were in each case proportionately much less active compared to *Rhodobacteraceae*, which showed in NA as well as in BA areas the second highest activities (NA = 6%, BS = 11%). Thus, the *Rhodobacteraceae* were the only family that had higher values based on RNA than DNA analyses in the NA and BS areas (Fig. 4).

### Relative abundance and activity of predominant OTUs in NA and BS areas.

The abundance of OTUs was analyzed using a model accounting for resampling from one individual and statistically comparing the difference in abundance between NA and BS areas. The 20 most abundant OTUs in both the NA and the BS samples are shown in Table 3 for RNA and DNA samples. A full list of OTUs with statistics is in (Supplemental File 3). The percentage of the 20 most abundant OTUs in NA areas was overall 34.36% of the total community. Here, the most abundant was OTU_6 (*Mycoplasma*), which made up 4.46%, and OTU_2 (*Loktanella*) had the lowest abundance with 0.82%. The percentage of the 20 most abundant OTUs in BS areas was much higher, i.e., 58.68% of the total community, with OTU_1 (*Aquimarina*) being the most abundant with 18.67%, and OTU_20 (Eel-36e1D6, Deltaproteobacteria) having the lowest abundance with 0.75%. In NA areas, 6 OTUs belonging to five classes were present at >2% (summed up to 18.81%). In BS areas, we found 8 OTUs >2% (summed up to 44.95%) and belonging to 4 classes (Table 3).

**TABLE 3:** Relative abundance (based on DNA) and activity (based on RNA) of the 40 predominant OTUs identified on C. pagurus, i.e., the 20 most abundant OTUs in nonaffected areas (NA) and 20 in black spot syndrome affected areas (BS) were considered^*a*^

20 most abundant OTUs in NA	Relative abundance (%) DNA		Wald test	Relative abundance (%) DNA		Wald test
	NA	BS		NA	BS	
*Alphaproteobacteria*						
OTU_2 (*Loktanella*)^*b*^	0.82^*b*^	2.95^*b*^	−4.17^*b*^^,^^*d*^	1.06^*b*^	5.82^*b*^	−5.43^*b*^^,^^*e*^
OTU_7 (*Robiginitomaculum*)	4.18	2.31	0.80	2.00	1.31	0.64
OTU_16 (*Ahrensia*)	1.64	0.77	1.13	1.66	0.71	0.67
OTU_15 (*Anderseniella*)	1.30	1.33	−0.61^*c*^	1.20	1.35	−1.67
OTU_25 (*Hyphomonadaceae*)	0.93	0.72	0.39	1.07	0.74	1.04
*Gammaproteobacteria*						
OTU_29 (*Xanthomonadales*)	0.99	0.38	0.66	0.62	0.19	1.42
OTU_24 (*Granulosicoccus*)	1.16	0.28	1.65	1.49	0.42	1.63
OTU_4^*a*^ (*Perspicuibacter*)	3.41^*b*^	1.09^*b*^	−2.61^*b*^^,^^*c*^	3.85^*b*^	1.18^*b*^	−3.90^*b*^^,^^*d*^
OTU_13 (*Thiotrichaceae*)	1.20	1.33	−0.34	0.57	0.88	−0.04^*c*^
OTU_60 (*Cellvibrionaceae*)	0.83	0	2.51	0.42	0	−0.99
*Flavobacteriia*						
OTU_19 (*Tenacibaculum*)	2.30	0.19	−0.22	0.87	0.11	−0.18
OTU_10 (*Maritimimonas*)	2.07	2.46	0.21	0.74	1.15	0.47^*c*^
OTU_37 (*Aquibacter*)	1.02	0.13	2.00	0.50	0.06	1.49
OTU_52 (*Maribacter*)	0.93	0.10	2.26	0.20	0.05	1.91
*Acidimicrobiia*						
OTU_14 (Sva0996 marine group)	2.39	0.58	1.79	2.09	0.73	1.26
OTU_21 (*Acidimicrobiales*)	1.54	0.44	1.53	1.40	0.55	1.33
*Nitrospira*						
OTU_18 (*Nitrospira*)	1.30	0.38	1.65	1.84	0.36	2.05
*Bacilli*						
OTU_41 (*Granulicatella*)	1.03	1.10	1.03	0.40	0.82	−0.36
*Sphingobacteriia*						
OTU_38 (*Portibacter*)	0.86	0.43	1.92	0.51	0.29	1.11
*Mollicutes*						
OTU_6 (*Mycoplasma*)	4.46	9.20	0.69	3.98	6.52	0
20 most abundant OTUs in BS	Relative abundance (%) DNA		Wald test	Relative abundance (%) DNA		Wald test
	NA	BS		NA	BS	
*Alphaproteobacteria*						
OTU_2 (*Loktanella*)^*b*^	0.82^*b*^	2.95^*b*^	−4.16^*b*^^,^^*d*^	1.06^*b*^	5.83^*b*^	−5.43^*b*^^,^^*e*^
OTU_8 (*Pelagibius*)	0.02	2.22	−4.69^*c*^	0.04	2.25	−5.28
OTU_7 (*Robiginitomaculum*)	4.18	2.31	0.80	2	1.31	0.64
OTU_17 (*Sulfitobacter*)	0.32	0.93	−1.94	0.44	0.85	−2.27
OTU_15 (*Anderseniella*)	1.30	0.78	−0.61	1.20	1.35	−1.67
OTU_16 (*Ahrensia*)	1.64	0.77	1.13	1.66	0.71	0.67
OTU_23 (Thalassobius)	0.62	0.96	−0.99	0.62	0.95	−0.43
*Gammaproteobacteria*						
OTU_3 (*Perspicuibacter*)	0.05	4.98	−3.13^*f*^	0.02	4.78	−4.07
OTU_4 (*Perspicuibacter*)^*b*^	3.41^*b*^	1.09^*b*^	−2.61^*b*^^,^^*c*^	3.85^*b*^	1.18^*b*^	−3.90^*b*^^,^^*d*^
OTU_5 (*Eionea*)	0	1.67	−0.90	0	1.09	−6.08
OTU_11 (*Cocleimonas*)	0.76	1.32	−0.74	0.37	0.89	0.04
OTU_12 (*Arenicella*)^*b*^	0.23^*b*^	1.38^*b*^	−4.17^*b*^^,^^*d*^	0.20^*b*^	1.01^*b*^	−4.87^*b*^^,^^*e*^
OTU_13 (*Thiotrichaceae*)	1.20	1.33	−0.34	0.57	0.88	−0.04
OTU_9 (*Cellvibrionaceae*)^*b*^	0.01^*b*^	2.16^*b*^	−6.43^*b*^^,^^*e*^	0^*a*^	2.12^*b*^	−11.47^*b*^^,^^*e*^
*Flavobacteriia*						
OTU_1 (*Aquimarina*)^*b*^	0.31^*b*^	18.67^*b*^	−6.86^*b*^^,^^*e*^	0.18^*b*^	14.51^*b*^	−7.29^*b*^^,^^*e*^
OTU_10 (*Maritimimonas*)	2.07	2.46	0.21	0.74	1.15	0.47
*Mollicutes*						
OTU_6 (*Mycoplasma*)	4.46	9.20	0.69	3.98	6.52	0
*Bacilli*						
OTU_41 (*Granulicatella*)	1.03	1.10	1.03^*b*^	0.40	0.82	−0.36
*Betaproteobacteria*						
OTU_68 (*Comamonadaceae*)	0.22	1.10	−1.17	0.26	1.17	−0.78
Deltaproteobacteria						
OTU_20 (*Eel-36e1D6*)	0.03	0.75	−1.55	0.03	2.84	−2.71

a Classification of OTUs is given on class level and lowest definable taxonomic level (in brackets).

b Significant changes in abundance based on Wald statistics.

c Significance for differences in abundance based on Wald statistic was not calculated due to violation of negative binomial distribution or low counts.

d *P* < 0.01, with the significance level after false discovery rate adjustment following Benjamini and Hochberg (53).

e *P* < 0.001, with the significance level after false discovery rate adjustment following Benjamini and Hochberg (53).

f *P* < 0.05, with the significance level after false discovery rate adjustment following Benjamini and Hochberg (53).

Most of the abundant OTUs found in NA areas belong to *Alphaproteobacteria* (5), *Gammaproteobacteria* (5), and *Flavobacteriia* (4). Compared to NA areas, more OTUs from *Alphaproteobacteria* (7) and *Gammaproteobacteria* (7) were found in BS samples (Table 3). Within the *Alphaproteobacteria*, the differences were OTU_8 *Pelagibius*, which was nearly missing in NA samples, and OTU_17 *Sulfitobacter* and OTU_23 *Thalassobius*, which were present and more abundant in BS samples than in NA samples. The strongest difference for *Alphaproteobacteria* was observed in OTU_2 *Loktanella*, which was three times more abundant in BS samples (2.95%) and almost six times more active based on RNA pools (5.82%), making it the third most active OTU in BS areas. Both increases were significant using the Wald test statistic (DNA, N = 8, *P* ≤ 0.01; RNA, N = 8, *P* ≤ 0.001; Table 3). Within the *Gammaproteobacteria*, the strongest differences were found in OTU_5 *Eione*, which was absent in NA samples but accounted for an average of 1.67% of OTUs in the BS samples. Other *Gammaproteobacteria* strongly increased in BS samples, such as OTU_12 *Arenicella* (six times), OTU_9 *Cellvibrionaceae* (216 times) or OTU_3 *Perspicuibacter* (100 times). On the other site, percentages of OTU_4 *Perspicuibacter* (NA = 3.41% versus BS = 1.09%, *P* = not applicable; Table 3) and OTU_60 *Cellvibrionaceae* (NA = 0.83% versus BS = 0%, *P* = not applicable; Table 3) were higher in NA samples, however, both changes were not significant for DNA samples.

Strong changes in relative abundance in DNA pools corresponding to RNA pools were also present in *Flavobacteria*. There were fewer OTUs with high abundance in BS samples, i.e., OTU_19 *Tenacibaculum*, OTU_37 *Aquibacter*, and OTU_52 *Maribacter* were almost completely absent in BS compared to NA pools. Contrarily, OTU_1 *Aquimarina* was nearly missing in NA samples but 60 times more abundant in BS samples, also showing the highest percentage in activity (14.51% on average), with high statistical significance using the Wald test (N = 8, Wald = −7.29, *P* ≤ 0.001).

### Isolation and phylogeny of new C. pagurus-associated strains.

**FIG 5:**
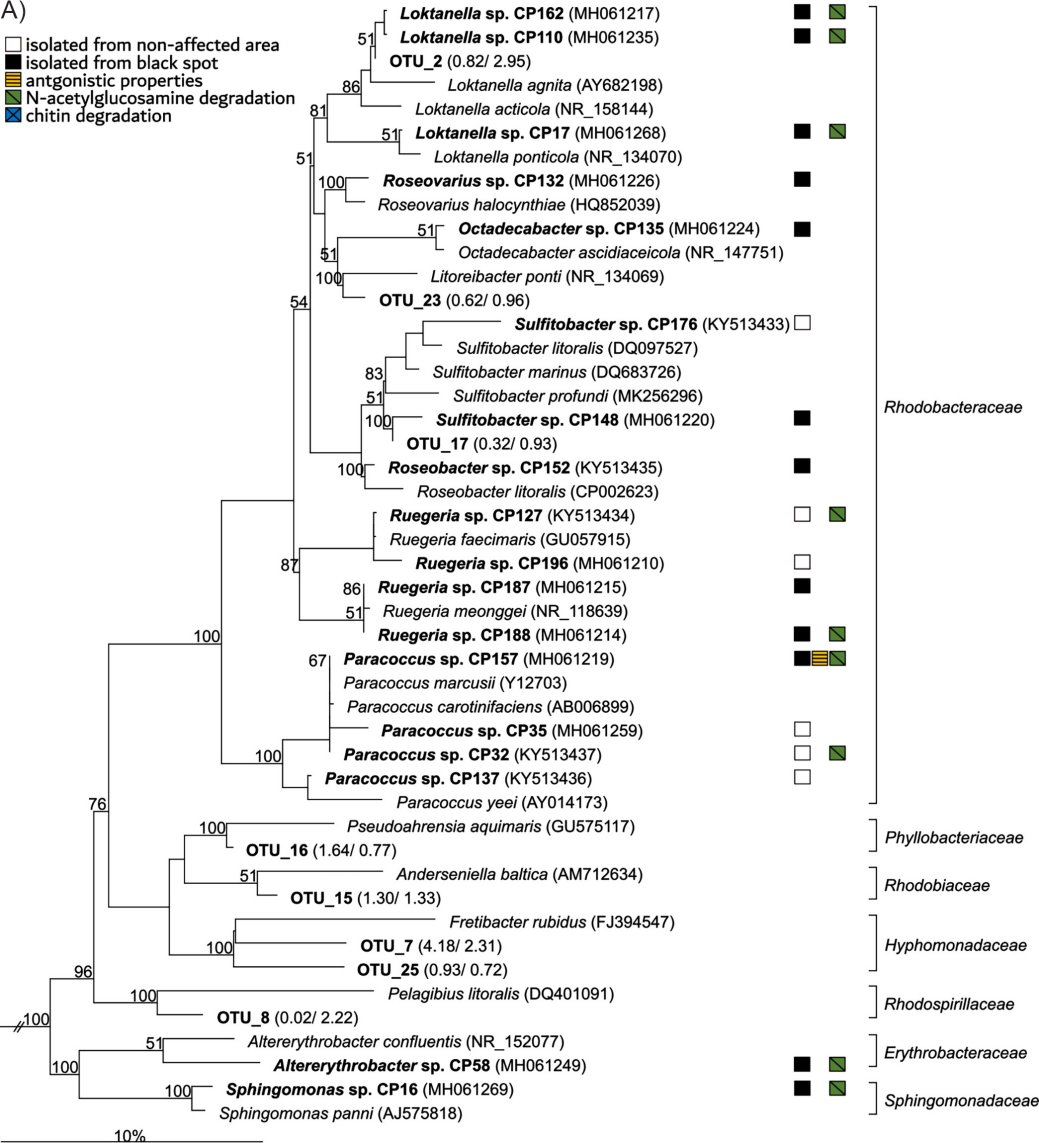

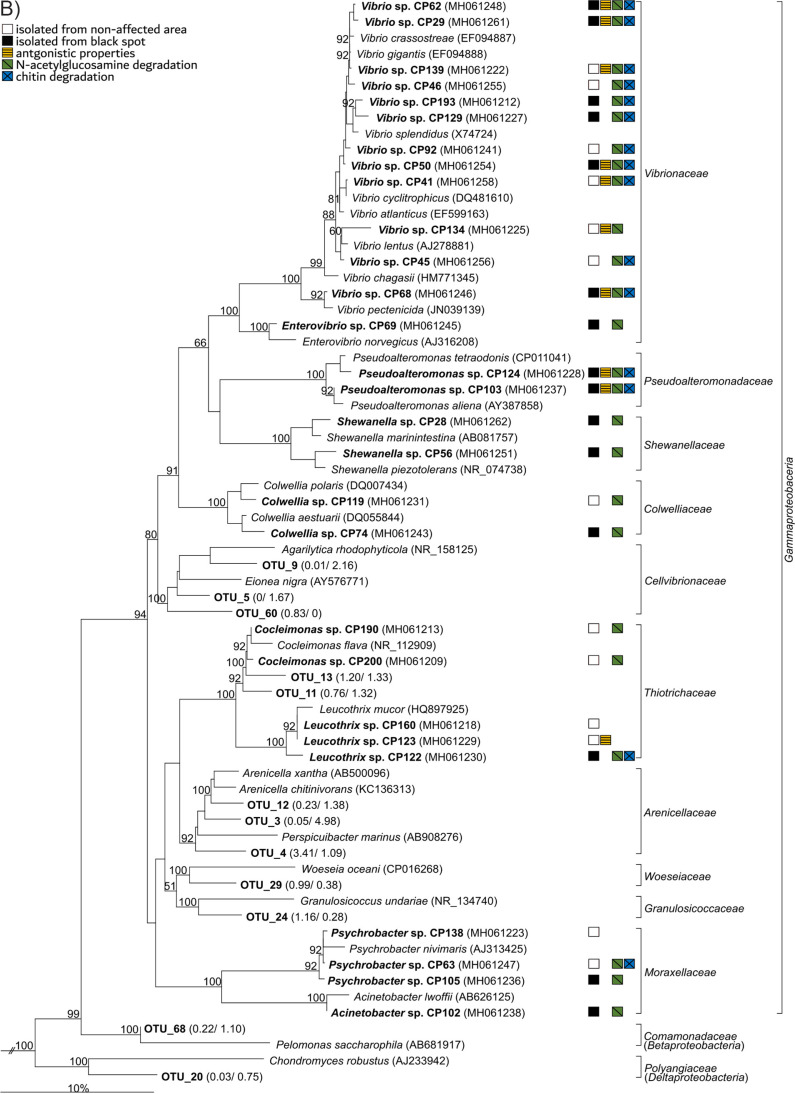

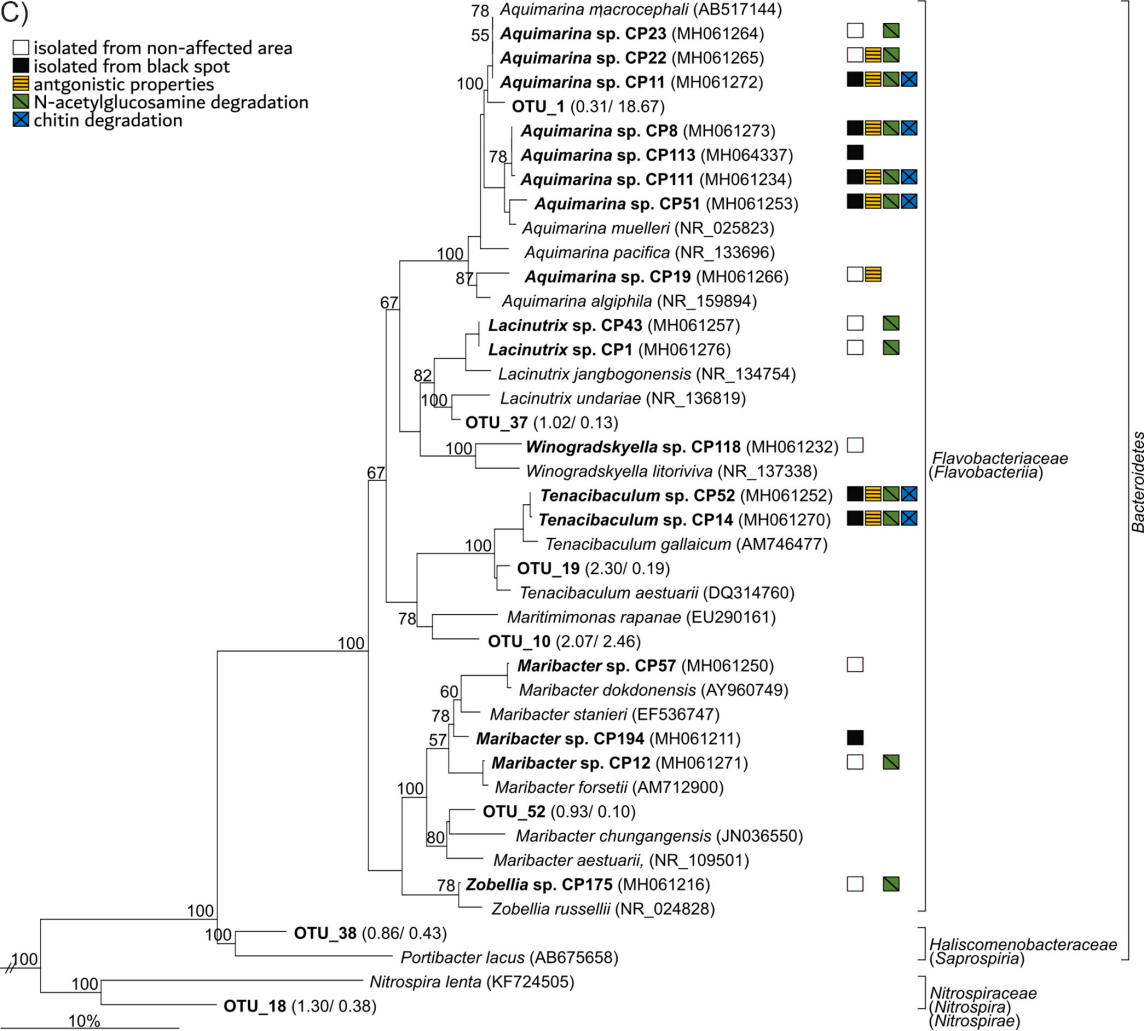

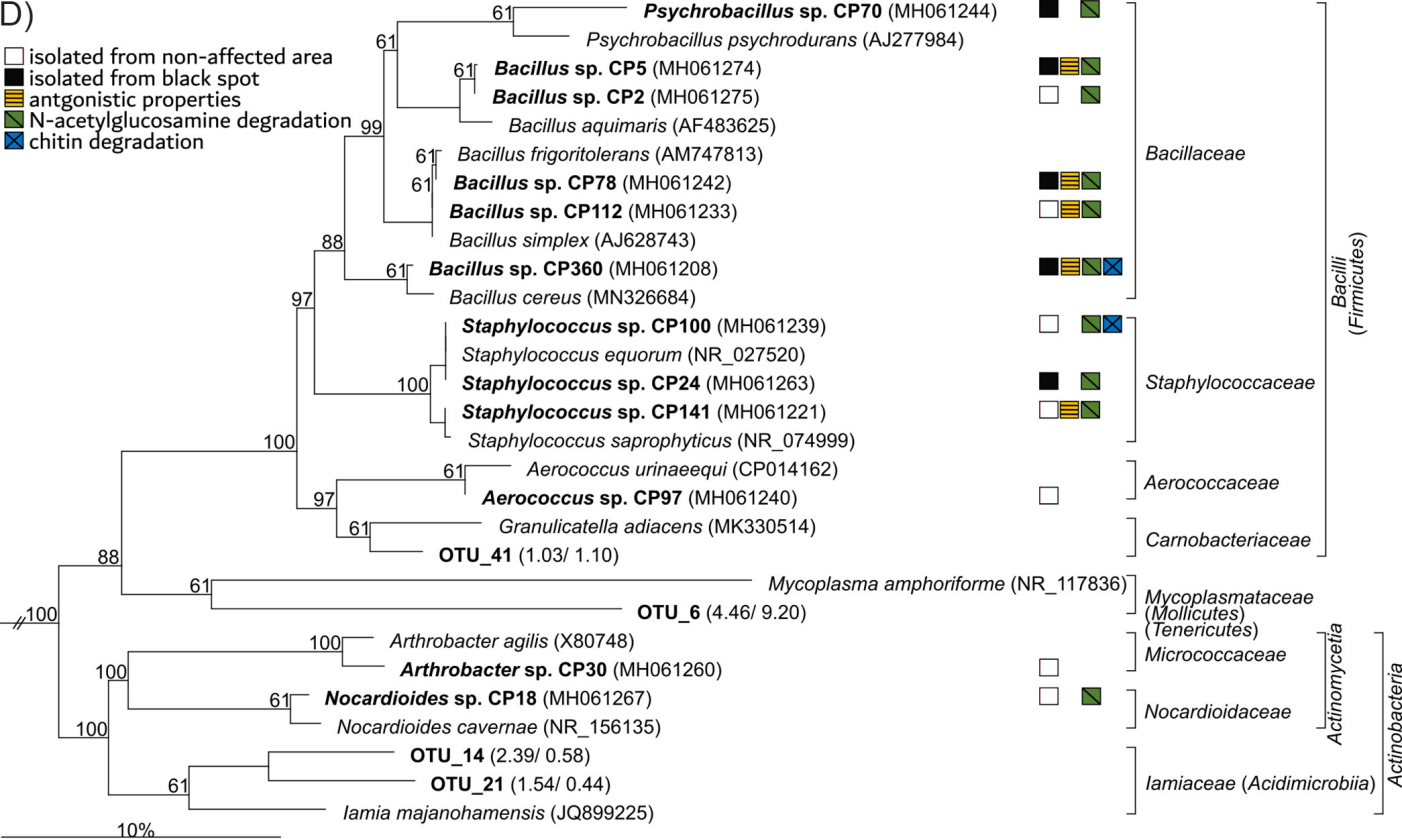
Neighbor-joining trees based on 16S rRNA gene similarity showing the phylogenetic affiliation of isolates and OTU consensus sequences obtained in this study (bold) within the *Alphaproteobacteria* (A), *Gammabacteria* and *Betaproteobacteria* (B), *Bacteriodetes* and *Nitrospirae* (C), *Firmicutes*, *Tenericutes*, and *Actinobacteria* (D). Only bootstrap values ≥50% (derived from 1000 replicates) for the main nodes are shown. Selected sequences related to *Cyanobacteria* were used as outgroup in order to define the root of the tree (not shown). GenBank accession numbers are given in parentheses. Scale bars indicate the percentage sequence divergence.

Using five different media, we isolated 75 different bacterial strains from the carapaces of the four C. pagurus specimens sampled in this study. From NA areas, 35 strains and from BS areas 40 strains were obtained. Strains belonging to the five classes *Gammaproteobacteria* (37.3%), *Alphaproteobacteria* (24%), *Flavobacteriia* (22.6%), *Bacilli* (13.3%), and *Actinobacteria* (2.7%) and affiliated with 14 families, with one-third of the strains belonging to either *Flavobacteriaceae* (17 strains) or *Rhodobacteraceae* (16 strains). At the genus level, 12 strains affiliated with *Vibrio*, eight with *Aquimarina*, five with *Bacillus,* and four each with *Paracoccus* and *Ruegeria* (Fig. 5, Table 4). Sixty strains were isolated with half strength MB medium (Difco marine broth [MB] 2216 [BD Biosiences, Franklin Lakes, NJ]), five with salt water (SWM) supplemented with peptone, four with purified chitin, four with peptone and chitin, and two with a pestled carapace of C. pagurus (Table 4). All *Alphaproteobacteria*, all *Flavobacteriia*, and most *Gammaproteobacteria*, except the *Vibrionaceae*, were isolated with half strength MB medium. Most *Vibrionaceae* were isolated with various media containing chitin, thus being the most versatile group concerning growth on the media used (Table 4). Isolates of the Gram-positive classes *Actinobacteria* and *Bacilli* were also obtained using a variety of media (Table 4).

**TABLE 4:** Taxonomic affiliation based on 16S rRNA gene sequences of new bacterial strains isolated from the carapace of Cancer pagurus specimens. Given are our source (NA = nonaffected or BS = black spot shell diseased areas), the closest described relative (accession number), the similarity of the respective 16S rRNA gene sequence, and the medium used for isolation

Class/family	Strain	Source	Closest described relative^*a*^	16S rRNA similarity (%)	Medium^*b*^
*Alphaproteobacteria*					
* Rhodobacteraceae*					
	CP132	BS	Aliiroseovarius halocynthiae MA1-10 (NR_109323)	98.9%	1/2 MB
	CP162	BS	Loktanella acticola OISW-6 (NR_158144)	98.1%	1/2 MB
	CP110	BS	Loktanella acticola OISW-6 (NR_158144)	98.1%	1/2 MB
	CP17	BS	Loktanella ponticola W-SW2 (NR_134070)	99.2%	1/2 MB
	CP135	BS	Octadecabacter ascidiaceicola RA1-3 (NR_147751)	99.5%	1/2 MB
	CP157	BS	Paracoccus carotinifaciens E-396 (NR_024658)	99.7%	1/2 MB
	CP35	NA	Paracoccus marcusii MH1 (NR_044922)	98.6%	1/2 MB
	CP32	NA	Paracoccus marcusii MH1 (NR_044922)	100%	1/2 MB
	CP137	NA	Paracoccus yeei G1212 (NR_029038)	100%	1/2 MB
	CP152	BS	Roseobacter litoralis Och 149 (NR_074143)	98.8%	1/2 MB
	CP127	NA	Ruegeria faecimaris HD-28 (NR_104546)	100%	1/2 MB
	CP196	NA	Ruegeria faecimaris HD-28 (NR_104546)	98.8%	1/2 MB
	CP188	BS	Ruegeria meonggei MA-E2-3 (NR_118639)	100%	1/2 MB
	CP187	BS	Ruegeria meonggei MA-E2-3 (NR_118639)	100%	1/2 MB
	CP176	NA	Sulfitobacter litoralis Iso 3 (NR_043547)	97.0%	1/2 MB
	CP148	BS	Sulfitobacter marinus SW-265 (NR_043936)	97.8%	1/2 MB
* Sphingomonadaceae *	CP58	BS	Altererythrobacter confluentis KEM-4 (NR_152077)	94.8%	1/2 MB
	CP16	BS	Sphingomonas panni C52 (NR_042193)	99.1%	1/2 MB
*Gammaproteobacteria*				
* Colwelliaceae*	CP74	BS	Colwellia aestuarii SMK-10 (NR_043509)	98.3%	1/2 MB
	CP119	NA	Colwellia polaris 539-7 (NR_043462)	98.7%	1/2 MB
* Moraxellaceae*	CP102	BS	Acinetobacter lwoffii JCM 6840 (NR_113346)	100%	1/2 MB
	CP138	NA	Psychrobacter nivimaris 88/2-7 (NR_028948)	99.7%	1/2 MB
	CP63	NA	Psychrobacter nivimaris 88/2-7 (NR_028948)	99.9%	1/2 MB
	CP105	BS	Psychrobacter nivimaris 88/2-7 (NR_028948)	99.5%	1/2 MB
*Pseudoalteromonadaceae*					
	CP103	BS	Pseudoalteromonas aliena KMM 3562 (NR_025775)	100%	1/2 MB
	CP124	BS	Pseudoalteromonas tetraodonis GFC (CP011041)	99.3%	1/2 MB
* Shewanellaceae *	CP28	BS	Shewanella marinintestina IK-1 (NR_024791)	99.7%	1/2 MB
	CP56	BS	Shewanella piezotolerans WP3 (NR_074738)	98.3%	1/2 MB
* Thiotrichaceae*	CP190	NA	Cocleimonas flava KMM 3898 (NR_112909)	100%	1/2 MB
	CP200	NA	Cocleimonas flava KMM 3898 (NR_112909)	99.4%	1/2 MB
	CP122	BS	Leucothrix mucor ATCC 25107 (NR_118099)	98.1%	1/2 MB
	CP123	NA	Leucothrix mucor ATCC 25107 (NR_118099)	99.7%	1/2 MB
	CP160	NA	Leucothrix mucor ATCC 25107 (NR_118099)	99.7%	1/2 MB
* Vibrionaceae*	CP69	BS	Enterovibrio norvegicus LMG 19839 (NR_042082)	99.3%	1/2 MB
	CP129	BS	Vibrio atlanticus VB 11.11 (NR_116067)	99.1%	P-SWM
	CP45	NA	Vibrio atlanticus VB 11.11 (NR_116067)	99.5%	CP-SWM
	CP29	BS	Vibrio crassostreae LGP 7 (NR_114909)	99.3%	CN-SWM
	CP92	NA	Vibrio crassostreae LGP 7 (NR_044078)	99.3%	1/2 MB
	CP50	BS	Vibrio chagasii LMG 21353 (NR_117891)	99.5%	1/2 MB
	CP41	NA	Vibrio cyclitrophicus LMG21359 (NR_115806)	99.9%	CP-SWM
	CP46	NA	Vibrio gigantis LGP 13 (NR_114910)	99.6%	AC-SWM
	CP62	BS	Vibrio gigantis LGP 13 (NR_044079)	99.4%	CN-SWM
	CP139	NA	Vibrio gigantis LGP 13 (NR_114910)	100%	CN-SWM
	CP193	BS	Vibrio splendidus ATCC 33125 (NR_119060)	99.2%	CP-SWM
	CP134	NA	Vibrio lentus 4OM4T (NR_028926)	98.5%	CN-SWM
	CP68	BS	Vibrio pectenicida LMG 19642 (NR_118241)	99.7%	AC-SWM
*Flavobacteriia*					
* Flavobacteriaceae*	CP19	NA	Aquimarina algiphila 9Alg 151 (NR_159894)	98.1%	1/2 MB
	CP11	BS	Aquimarina macrocephali JAMB N27 (NR_112971)	100%	1/2 MB
	CP23	NA	Aquimarina macrocephali JAMB N27 (NR_112971)	100%	1/2 MB
	CP22	NA	Aquimarina macrocephali JAMB N27 (NR_112971)	100%	1/2 MB
	CP8	BS	Aquimarina muelleri KMM 6020 (NR_025823)	98.8%	1/2 MB
	CP111	BS	Aquimarina muelleri KMM 6020 (NR_025823)	98.5%	1/2 MB
	CP113	BS	Aquimarina muelleri KMM 6020 (NR_025823)	98.8%	1/2 MB
	CP51	BS	Aquimarina muelleri KMM 6020 (NR_025823)	98.4%	1/2 MB
	CP43	NA	Lacinutrix jangbogonensis PAMC 27137 (NR_134754)	98.0%	1/2 MB
	CP1	NA	Lacinutrix jangbogonensis PAMC 27137 (NR_134754)	98.0%	1/2 MB
	CP57	NA	Maribacter dokdonensis DSW-8 (NR_043294)	99.7%	1/2 MB
	CP12	NA	Maribacter forsetii KT02ds18-6 (NR_042627)	99.7%	1/2 MB
	CP194	BS	Maribacter stanieri KMM 6046 (NR_116052)	98.7%	1/2 MB
	CP14	BS	Tenacibaculum gallaicum A37.1 (NR_042631)	99.5%	1/2 MB
	CP52	BS	Tenacibaculum gallaicum A37.1 (NR_042631)	99.6%	1/2 MB
	CP118	NA	Winogradskyella litoriviva KMM 6491 (NR_137338)	97.3%	1/2 MB
	CP175	NA	Zobellia russellii KMM 3677 (NR_024828)	99.9%	1/2 MB
*Actinobacteria*					
* Nocardioidaceae*	CP18	NA	Nocardioides cavernae YIM A1136 (NR_156135)	99.2%	1/2 MB
* Micrococcaceae*	CP30	NA	Arthrobacter agilis DSM 20550 (MN080900)	98.3%	P-SWM
*Bacilli*					
* Bacillaceae*	CP2	NA	Bacillus aquimaris TF-12 (NR_025241)	99.1%	P-SWM
	CP360	BS	Bacillus cereus ATCC 14579 (MN326684)	97.4%	CP-SWM
	CP78	BS	*Bacillus frigoritolerans* DSM 8801 (MK424281)	100%	1/2 MB
	CP112	NA	Bacillus simplex NBRC 15720 (CP017704)	100%	P-SWM
	CP5	BS	Bacillus aquimaris TF-12 (NR_025241)	99.0%	1/2 MB
	CP70	BS	Psychrobacillus psychrodurans 68E3 (NR_025409)	96.1%	1/2 MB
* Aerococcaceae*	CP97	NA	Aerococcus urinaeequi CCUG28094 (CP014162)	99.9%	1/2 MB
* Staphylococcaceae *	CP100	NA	Staphylococcus equorum PA 231 (NR_027520)	100%	P-SWM
	CP24	BS	Staphylococcus equorum PA 231 (NR_027520)	100%	1/2 MB
	CP141	NA	Staphylococcus saprophyticus ATCC 15305 (NR_074999)	100%	1/2 MB

a Affiliation identified by BLAST analysis (http://blast.ncbi.nlm.nih.gov/Blast.cgi).

b Abbreviations for used mediums: 1/2 MB, Marine Broth 2216 with 50% peptone and yeast extract; P-SWM, salt water medium^44^ supplemented with 0.1% (w/v) peptone; CP-SWM, SWM supplemented with 0.2% (w/v) purified chitin and 0.01% (w/v) peptone; AC-SWM, SWM supplemented with 0.2 autoclaved pisteled carapace material from Cancer pagurus; CN-SWM, SWM supplemented with 0.1% (w/v) purified chitin and 0.01 (w/v) *N*-acetylglucosamine.

### Physiological properties of bacterial strains.

In total, 23 strains (31%) were able to degrade chitin. Most chitin degraders were *Gammaproteobacteria* (15 strains), mainly members of the *Vibrionaceae* (11 strains), while none of the *Alphaproteobacteria* showed this ability. Furthermore, six *Flavobacteriia* and two strains of the *Bacilli* could degrade chitin (Fig. 5A to D). Seven chitin-degrading strains were isolated in NA areas and 16 from BS areas, thus the proportion of isolates capable of degrading chitin from BS areas (40%) was more than twice as high as that from NA areas (17%). Four out of five of our *Aquimarina* strains from BS were capable to degrade chitin. However, none of the three *Aquimarina* strains were isolated from NA areas (Fig. 5C). For the *Vibrionaceae* this trend was not visible, i.e., chitin degraders were obtained from NA and BS areas in almost equal numbers (Fig. 3B). The chitin monomer *N-acetylglucosamine* was used by 43 strains (57%), 17 isolates from NA, and 26 isolates from BS areas (Fig. 5). The fact that 20 of these strains cannot degrade chitin indicates that they can take advantage from the extracellular chitinase activity of the chitin degraders.

In total, 24 strains showed antagonistic activity against laboratory standard strains and strains obtained from C. pagurus. The inhibition spectra of most strains were unique, ranging between one and 18 inhibited target organisms (Table S5 in Supplemental File 1). More than two-thirds of the antagonistic isolates belong to genera *Vibrio*, *Aquimarina,* and *Bacillus* with seven, six, and four strains, respectively (Fig. 5). While most of these strains inhibited several target organisms, the broadest inhibition spectrum was observed for *Tenacibaculum* CP14 and CP52, which inhibited 18 and 14 test strains, respectively. *Vibrio* CP68 inhibited 13 target strains and showed the largest inhibition zones. Seven strains from NA areas and 17 strains isolated from BS showed antimicrobial effects against laboratory target strains. Target strains from C. pagurus were inhibited by 13 isolates, most of which also inhibited our standard target organisms and were mainly affiliated with *Vibrio*, *Aquimarina*, *Bacillus,* and *Tenacibaculum* (Table S5 in Supplemental File 1).

### Comparison of bacterial isolates with OTUs.

Overall, the 16S rRNA gene sequences of 39 out of 75 bacterial isolates obtained in this study matched with those of 96 OTUs of the MiSeq data with ≥97% similarity. Twelve of these isolates show even 100% sequence similarity to OTUs (Table S4 in Supplemental File 1). Phylogenetic analysis of the new strains and the 20 most abundant OTUs from NA and BS areas (Table 3) demonstrates that in some cases even sequences of these OTUs and strains are closely related with sequence similarities ≥97%. This is the case for *Loktanella* strains CP162 and CP110 and OTU_2 (99.5% similarity each), *Sulfitobacter* CP148 and OTU_17 (100% similarity) (Fig. 5A), *Cocleimonas* CP200 and OTU_13 (97.2% similarity) (Fig. 3B), *Aquimarina* strains CP11, CP22, CP23 and CP8, CP111, and CP113 with OTU_1 (98.6% similarity each and 97.2% similarity each, respectively), *Tenacibaculum* strains CP14 and CP52 and OTU_19 (97.6% similarity each), and *Lacinutrix* CP1 and CP43 and OTU_37 (97.4% similarity each) (Fig. 5C). Thus, three OTUs (OTU_1, OTU_2, and OTU_17), with a sequence similarity of ≥ 98.5% to six isolates, were found in increased abundance in BS affected regions (Table 3). Especially physiological data of the closely related strains can be used to complement the information derived from the abundant OTUs.

## DISCUSSION

We described, for the first time, the microbiome of NA areas in direct comparison to BS-affected areas of the carapace of C. pagurus, elucidating a shifted and reduced microbial diversity in diseased areas toward an increased abundance of *Flavobacteriaceae* and *Rhodobacteraceae*. The physiological experiments with bacterial strains enabled us to discuss the characteristics of our isolates in context with the microbial community composition, suggesting a high level of adaption, interactions, and resistance capacities of members originating from the same habitat. Furthermore, our data indicate a common, even, and thus stable core community in NA samples. Epibacterial communities in BS areas are subject to fluctuations and changes in composition due to changes in the abundance of preexisting genera that are also present in NA areas rather than invasion by external genera (Table 3). Community analysis based on RNA sequences showed a similar trend (Table 1), although DNA-derived communities were generally less diverse and less even than RNA-derived communities. The dissimilarity between DNA and RNA supports the hypothesis that RNA is probably more indicative of active members of investigated communities and underlines that stand-alone 16S rRNA gene results should be interpreted cautiously (20). Nevertheless, the DNA and RNA-derived microbial communities overlapped; of the abundant OTUs, none was found exclusively in either the DNA or RNA data set, indicating that the abundant bacteria constitute active members of the communities (Table 3).

The microbial composition of BS was characterized by a high abundance of *Flavobacteria*, *Alphaproteobacteria,* and *Gammaproteobacteria*. Within the *Flavobacteriia*, the genus *Aquimarina* (*Flavobacteriaceae*) was detected in BS, especially the *Aquimarina-*affiliated OTU_1, which was present in almost every investigated BS (16 of 17). Overall, 12 OTUs were assigned to *Aquimarina*, of which OTU_1 was the most abundant member of the *Flavobacteriia* within BS representing, on average, 18.67% of the total community in comparison to 0.31% relative abundance in NA. Moreover, the transcriptional activity (RNA) of OTU_1 increased on average from 0.18 to 14.51% in BS (Table 3). *Aquimarina* spp. have previously been found associated with planktonic algae, crustaceans, or marine sponges (21). Some members of *Aquimarina* were reported to be opportunistic pathogens in *Rhodophyta* and are known to enzymatically hydrolyze marine polysaccharides like chitin, the major component of the exoskeleton of crustaceans (21–23). *Aquimarina* sp. I32.4 was identified as a potential pathogen in the development of shell disease in Homarus americanus due to its enzymatic activities on lipids, proteins, and chitin components of the lobster carapace (19). The *Aquimarina* strains CP11, CP22, and CP23 isolated in this study showed 98.6% genetic similarity to the most abundant amplicon OTU_1 in BS areas (Table S4 in Supplemental File 1). Remarkably, OTU_1 (422 bp) had 100% genetic similarity to the 16S rRNA gene sequence within the genome of *Aquimarina* sp. I32.4 (PGUB01000172.1), which was described in the context of the epizootic shell disease of the American lobster (19). Of our eight *Aquimarina* isolates, six showed antagonistic behavior, of which five were obtained from BS areas. The antimicrobial potential of members of *Aquimarina* spp. could contribute to the increased abundance of this genus in BS and may lead to a progression of black spot areas, favoring chitin degraders in the bacterial community, including *Aquimarina* spp. (Fig. 5C).

Also belonging to the *Flavobacteriaceae*, the genus *Tenacibaculum* is associated with the black spot shell disease syndrome (16, 24). OTU_19 is affiliated with this genus and was among the 20 most abundant OTUs in NA areas. *Tenacibaculum* also comprises pathogens like Tenacibaculum maritimum (formerly Flexibacter maritimus), which has been reported to be the etiological agent of tenacibaculosis in marine fish (25–27). Tenacibaculum discolor and Tenacibaculum gallaicum were described to be bacteriolytic (28). We could isolate one member of the *Tenacibaculum*, strain CP14 (closest relative T. gallaicum) that accordingly showed antagonistic behavior against 11 out of 17 isolates from C. pagurus and seven out of nine internal standard background strains (Table S5 in Supplemental File 1). Despite this, the genus *Tenacibaculum* varied in abundance in our data set and was found in lower abundance in BS and, thus, seems to be only loosely connected to shell disease syndrome.

Members of the *Rhodobacteraceae* are known to be associated with various marine eukaryotes, including sponges and algae (29, 30). Organisms of this family display a wide range of metabolic capabilities, including the production of antibiotic compounds against algae and bacteria, various toxins, aromatic compound degradation, carbon monoxide oxidation, or sulfur transformation (31). Within the *Rhodobacteraceae*, two known epibionts of the marine Rhodophyta Delisea pulchra, i.e., Nautella italica R11 and *Phaeobacter* sp. LSS9 were shown to cause symptomatic bleaching in algal spore-lings during an *in vitro* infection assay (32), indicating that these bacteria have the potential to cause pathogenic changes in epibacterial communities. Members of the *Rhodobacteraceae* could benefit from their metabolic physiological diversity triggering the degradation of different complex substrates. Their antagonistic properties against other microbes may give them additional advantages in the face of changes in the microbial community, such as those caused by black spot disease. It is known that many species affiliated with the *Roseobacter* group, i.e., the marine *Rhodobacteraceae* (33), produce secondary metabolites, including antibiotic compounds against other marine bacteria and algae, and various toxic substances, such as okadaic acid and paralytic toxins (31). One of the most abundant OTUs in BS areas, OTU_2, which is affiliated with the genus *Loktanella*, was detected in 13 out of 17 black spots on all specimens. OTU_2 was also among the most abundant OTUs in NA areas. However, its abundance increased in BS areas, accompanied by an increase of rRNA, indicating an active role in black spot shell disease.

The genus *Eionea* and OTU_9 (both *Cellvibrionaceae* and *Gammaproteobacteria*) were one of the few examples with higher abundance and increased activity in BS, but nearly absent in NA areas (Table 3, Supplemental File 3). The family of *Cellvibrionaceae* includes saprophytic species and complex polysaccharide degrading bacteria (34). Their capability to degrade chitin, cellulose, or agarose provides them a selective advantage to occupy distinct ecological niches, for instance, the abraded carapace of C. pagurus, and suggests that *Cellvibrionaceae* act as opportunistic colonizers in BS. Another dominant family of the *Gammaproteobacteria* found on C. pagurus, the *Arenicellaceae* (Fig. 4), was represented by 19 OTUs, including three OTUs affiliated with the most abundant genera in black spots, i.e., *Arenicella* sp. OTU_12, and *Perspicuibacter* sp. OTU_3 and OTU_4. Although we could not cultivate an isolate from this family, the amplicon data showed a higher abundance in BS associated with a higher RNA content. *Arenicella* spp. have been reported to be among the first colonizers in experimentally caused shell lesion communities of lobsters, indicating their possible role in initiating necrosis (3). *Perspicuibacter* was found in 12 out of 17 BS on each specimen. Overall, OTU_4 (*Perspicuibacter*) showed the highest value for relative abundance, but high variation between samples. One possible explanation for this increased variation could be that the development of the black spot syndrome goes through different stages, associated with temporal variations in the composition of the microbial community. Feinman et al. (18) observed *Aquimarina*, *Loktanella*, *Perspicuibacter,* and *Arenicella* spp. in shell lesions of laboratory-reared American lobster H. americanus and reported a significant reduction and loss of bacterial diversity in the shell disease-affected areas. According to this result, our observed reduction in microbial diversity on C. pagurus can be interpreted as microbial dysbiosis. This is interesting in context with the findings of Meres et al. (16) for epizootic shell disease of H. americanus. They argue that this kind of shell disease is caused by a polymicrobial shift, a dysbiosis rather than changes caused by a single strain.

Extracellular chitin degradation, observed in 31% of the isolated strains (i.e., 20% from nonaffected regions/40% from black spots) leads to the extracellular formation of *N-*acetylglucosamine. However, even for those strains lacking the ability to degrade chitin, this can indirectly also be utilized by organisms that can grow on the monomer, a characteristic found for most of our strains (75% of all isolates, 66% from NA, and 83% from BS areas). Besides knowing the selection bias of cultivation methods, the high occurrence of the abilities for chitin degradation and its monomer (Fig. 5) could be interpreted as bio-ecological coherence and bacterial interaction as well as the interaction between bacteria and their host. Because degradation of chitin is one major process leading to the shell disease syndrome of crustaceans (10, 11, 19), our findings contribute to the picture of what changes in epibacterial communities on carapaces and what specific bacterial strains are related to shell disease in C. pagurus.

Seven strains from NA and 17 strains from BS showed antimicrobial properties against standard target strains while 13 strains had antagonistic properties against other strains isolated from the carapace of C. pagurus. These results could indicate a higher abiotic efficiency against outsider strains, in comparison to a lower susceptibility of isolates from the same habitat. This supports the hypothesis of Cordero et al. (35) that acquired resistance could be a common trait in a host-specific community. Antimicrobial substances could constitute public goods within bacterial communities, benefiting non-producing but resistant community members in the epibacterial community on C. pagurus (35). However, for the majority (70%) of strains obtained from black spots antagonistic properties were observed (Fig. 5; Table S5 in Supplemental File 1), this feature might also contribute to the shifted and reduced bacterial community.

Although in our study the genus *Vibrio* was found in low abundance, the capability of the isolates to degrade chitin, in combination with their production of antagonistic compounds, could let them alter the microbial composition of C. pagurus and be harmful to the host. For this genus, we obtained the highest number of strains in the isolation approach. Among those, we found *Vibrio* sp. CP68 was closely related to Vibrio pectenicida, which is a pathogen in scallops (36) (Table S4 in Supplemental File 1). Wietz et al. (37) proved that chitin stimulates the production of the antibiotic andrimid in a Vibrio coralliilyticus strain. Thus, in an advanced state of the black spot syndrome, accompanied by the loss of the chitin protecting epicuticle, accessibility of chitin could lead to higher production of antibiotics like andrimid, leading to a repression of other strains, as assumed for other taxa discussed above.

The data from our culture-independent approach in combination with our cultivation experiments suggest that the black spot shell disease syndrome in C. pagurus is, besides other cofactors, not caused by a single pathogen, but by a microbial dysbiosis (17). A decrease in diversity could lead to a loss of functional redundancy in the microbiota. Theoretical models and laboratory experiments also suggest that the loss in eukaryotic host-associated microbial diversity, accompanied by changes in genetic and functional diversity, correlates with an increased disease risk under certain conditions, e.g., host competence - the ability to transmit diseases (38–42). The loss in diversity, accompanied by a functional loss of the microbiota, could therefore negatively affect the conditions of the host, and increase the susceptibility to the black spot shell disease syndrome. From our comparison of diseased and not diseased spots on the same C. pagurus specimen, we concluded that normally occurring host-associated bacteria emerge as potential opportunistic pathogens that alter the microbial interaction in the carapace biofilm on C. pagurus (17). Such shifts in diversity may happen due to apparent environmental stresses as we have shown earlier (17). Leinberger et al. (20), who studied the epibacterial community of the deep-sea galatheid Munidopsis alvisca, did not observe lesions or other morphological changes in the carapace that would indicate carapace disease in any of these animals from 2000 m depth. However, shell disease might be a normal phenomenon in crustaceans, possibly occurring also without anthropogenic influences or impacts of climate change (43). To target specific risks for crustaceans, broader approaches in combination with environmental modeling are necessary to understand whether bacterial community dysbiosis becomes problematic for species such as C. pagurus in their range of distribution and context of climate change.

## MATERIALS AND METHODS

### Capture and sampling of C. pagurus.

To study the epibacterial community composition associated with the carapace of C. pagurus, samples were collected on October 15th, 2014, along the northern coast of the island of Helgoland, southern North Sea. Four intermoult males of C. pagurus were captured in cages at a depth of 6.5 m at N 54°12.084” E 7°52.973” and N 54°12.097” E 7°52.971”. Animals were separated from each other and kept in clean plastic boxes with the wet ground (natural seawater, 15°C, 35‰ salinity). The carapace size of the crabs ranged between 15.5 to 17.0 cm (see Fig. S1 in Supplemental File 1). As a reference for bacterial community analysis (see below), three sterile 5 L Nalgene bottles were filled with seawater from the same location (15°C, pH 8.25, 35‰ salinity) and transported to the lab. Seawater was filtered through 0.2 μm polycarbonate membranes (47 mm, Whatman), and stored in sterile 2 mL Eppendorf tubes at −80°C until further processing. Animals were transported at 15°C within 1 h to the laboratory and washed three times with sterile filtered autoclaved artificial saltwater medium (SWM) (44) to remove loosely attached bacteria. Afterward, the animals were used directly for the sampling procedure. In total, 32 swab samples (Copan, Brescia, Italy, diameter <2 mm) were taken selectively from nonaffected areas (NA) and black spot syndrome affected areas (BS) with a diameter >2 mm. From each of the four specimens, three to five samples were taken from nonaffected spots (total N = 15) and black spot syndrome affected spots (total N = 17). Samples from NA spots were taken at a distance of at least 1 cm to BS spots. Samples for molecular biological analyses were frozen immediately in liquid nitrogen and stored at −80°C until further use. The sampling procedure for the isolation of bacterial strains sees below.

### DNA/RNA extraction and amplicon sequencing.

DNA and RNA from NA and BS areas were isolated with the AllPrep DNA/RNA Micro kit (Qiagen, Wolfenbüttel, Germany). We used the 32 swab samples for amplicon sequencing of the extracted DNA and RNA, resulting in an overall analysis of 67 samples, i.e., 64 samples from the C. pagurus specimens (32 samples for DNA as well as RNA-based analysis) and DNA of three water samples. DNA and RNA extracts were tested for quality and purity using a Nanodrop 2000s and a Qubit 2.0 Fluorometer. RNA samples were treated with RNase-free DNase (Qiagen), according to the manufacturer's instructions, and tested for remaining DNA by PCR and DNA measurements (Nanodrop 2000s). RNA samples were transcribed in cDNA using the AffinityScript RNA Synthesis kit (Agilent Technologies, Santa Clara, CA, USA). Further laboratory steps for MiSeq analysis were processed by LGC Genomics GmbH (Berlin, Germany). Amplicon libraries were prepared and individually barcoded. For all 67 samples, the hypervariable region V3 to V4 of the 16S rRNA gene was amplified using the barcode labeled primers 341F (5′-CCTACGGGNGGCWGCAG-3′) and 785R (5′-GACTACHVGGGTATCTAAKCC-3′) (45). Illumina libraries were constructed using the Ovation Rapid DR Multiplex System 1–96 (NuGEN Technologies, Inc., San Carlos, CA, USA) followed by MiSeq sequencing (Illumina, San Diego, CA, USA). For details, see Supplemental File 1.

### Demultiplexing and statistical analysis.

From a total of 67 samples, 2.5 million 300 bp paired-end reads were sequenced in two separate libraries using the Illumina MiSeq V3 platform. Demultiplexed and combined reads for each of the 67 samples were then analyzed using the LOTUs pipeline version 1.512, including quality filtering by sdm 1.27 beta (46). High-quality reads were clustered *de novo* by the UPARSE algorithm (47) with 99% similarity. Each OTU was assigned by blast algorithm (48) against the SILVA SSU Ref 99 version 132 database as reference (49) from phylum to species level. Statistical analysis was performed with Qiime 1.9.0 (50). Alpha diversity was calculated based on Chao and phylogenetic diversity. Differences between alpha diversity between treatments were analyzed using R version 3.2.3 (51) based on the Shannon-Wiener index and Simpson evenness calculated as inverse Simpson index divided by species richness using Qiime 1.9.0 (50). Different plots included in the Qiime 1.9.0 pipeline (50), such as PCoA, were used to visualize diversity among samples. The difference in alpha diversity between BS and NA areas of each specimen was tested using 2000 rarefied reads per pool.

The relative abundance of OTUs was calculated as an average over all samples from one individual and, finally, as an average for each condition (BS and NA) for either DNA or RNA samples (Table 3). To analyze the effect of BS versus NA condition of the carapace on the microbial community, the package DESeq2 (package version: 1.26.0 (52) was used to calculate a general linear model of the OTU counts based on a negative binomial distribution considering the samples from each individual in a model for paired data. DESeq2 transforms fold changes into logarithmic fold changes and uses several steps to shrink the log fold changes in cases of low counts, missing information, or outliers. The Cooks distance was used for testing the negative binomial distribution for each OTU and the independent filtering parameter was applied to cut off OTUs with few overall counts as described in Love et al. (52)). The Wald test as provided by DESeq2 was used for testing on significance in differential abundance between treatments. False discovery rate correction was done following Benjamini and Hochberg (53).

### Isolation of bacterial strains.

Samples taken from the carapace of C. pagurus specimens were directly transferred to agar plates containing five different media: (i) 1/2 marine broth (MB) (see Table S1 in Supplemental File 1), (ii) saltwater medium (SWM) after Zech et al. (44) supplemented with 0.1% (wt/vol) peptone, (iii) SWM supplemented with 0.2% (wt/vol) purified chitin and 0.01% (wt/vol) peptone, (iv) SWM supplemented with 0.2 (wt/vol) autoclaved and pestled carapace material of C. pagurus, and (v) SWM supplemented with 0.1% (w/V) purified chitin and 0.05 mM *N-*acetylglucosamine. All media contained 20 g L^−1^ Difco noble agar. Plates were incubated at 15°C for 6 weeks in the dark and single colonies were selected and transferred at least three times on the respective media until considered pure.

### 16S rRNA gene sequencing and phylogenetic analysis.

Isolates with similar colony morphologies were compared using denaturing gradient gel electrophoresis (DGGE) according to Teske et al. (54). The 16S rRNA genes of the isolates were amplified according to Brinkhoff and Muyzer (55), and PCR products were sent to Macrogen (Seoul, South Korea) for sequencing using the primers GM3f (56) and 1492R (57). The length of the obtained 16S rRNA gene fragments was between 588 and 1510 bp. Strains were considered for this study if they had differences in their 16S rRNA gene sequences. Strains with highly similar or identical sequences were also considered if they were obtained from both, BS or NA areas, or if they showed differences in their tested physiological properties (see below). Phylogenetic trees with 16S rRNA gene sequences obtained from the amplicon-based community analysis and the bacterial isolates were constructed using the ARB software package (www.arb-home.de (58–59)). Selected sequences of type strains related to Cyanobacteria were used as an outgroup (AP009552, FR798927, AF132782, AF115268, and EU078507). A backbone tree was calculated with nearly full-length 16S rRNA gene sequences (>1300 bp), and shorter sequences were added afterward with maximum parsimony.

### Growth experiments and physiological tests.

The growth of isolates was tested in SWM supplemented with 10 mM *N-*acetylglucosamine and monitored using a spectrophotometer (photoLab 6600 UV-VIS, WTW, Weilheim, Germany) over 4 weeks. For testing chitin degradation, chitin from crustaceans (Sigma, Aldrich) was prepared according to Hsu and Lockwood (60) and added to the SWM medium, including 20 g/L noble agar (Difco), to a final concentration of 0.1% (wt/vol) of purified colloidal chitin. Clearing zones of colloidal chitin formed by extracellular chitinase activity were monitored over 8 weeks.

### Screening for antagonistic effects.

Screening for antagonistic effects of isolates was performed with a modified Kirby-Bauer agar diffusion test (61). Each isolate was tested against nine target strains, affiliated with five different classes, and additionally against 17 strains also isolated from the carapace of C. pagurus and affiliated with five different classes (see Table S5 in Supplemental File 1). Before the screening, strains were cultivated for one to 5 days in MB 2216 medium at 15°C. Cultures of target strains were adjusted to an optical density at 600 nm (OD_600_) of 0.05, and cultures of isolates were tested to an OD_600_ of 0.2. Subsequently, a 200 μL culture solution of a target strain was pipetted on an agar plate and spread out with a Drigalski spatula. Afterward, test flakes (Carl Roth GmbH & Co. KG) were placed on the agar plate and each inoculated with 8 μL of liquid culture of the respective strain to be tested. Inhibition indicated by a bacteria-free zone (inhibition zone) was determined as the diameter (in millimeters) of the inhibitory zone between the paper disk and the bacterial lawn of the target strain. Tests were performed in triplicates and examined over 30 days. Isolates were considered antagonistic if they produced clearance zones >2 mm.

### Data availability.

Sequences of bacterial isolates obtained in this study were deposited at GenBank under the accession numbers KY513433-KY513437 (62), MH061208-MH061276, and MH064337. Sequences for each sample from the amplicon sequencing approach were uploaded to the European Nucleotide Archive (ENA) and can be found under the accession PRJEB40004. Under the same accession, OTUs are calculated based on 99% similarity among reads, and further analysis details are deposited.
