# Supplementary material for: Shell Disease Syndrome Is Associated with Reduced and Shifted Epibacterial Diversity on the Carapace of the Crustacean Cancer pagurus
Source: Microbiol Spectr. 2022 Nov 7;10(6):e03419-22. doi: 10.1128/spectrum.03419-22 (PMC9769784; doi:10.1128/spectrum.03419-22)
Supplement: Supplemental file 1 — Tables S1 to S5, Fig. S1, and Text S1. Download spectrum.03419-22-s0001.pdf, PDF file, 0.3 MB [file spectrum.03419-22-s0001.pdf]

Supplemental Material

Table S1 Composition of ½ MB (marine broth medium) used in this study. For solid medium 20 g L^{-1} agar were added. The medium was boiled up and 10 ml of trace elements (Table S2) were added before autoclaving.

Compound	g/L
Pepton	2.5
Yeast extract	0.5
Iron III citrate	0.1
$MgCl_2x6H_2O$	12.6
Na_2SO_4	3.24
NaCl	19.45
$CaClx2H_2O$	2.38
KCl	0.55
$NaHCO_3$	0.16
Na_2HPO_4 $2H_2O$	0.01

Table S2 Composition of trace element solution used in this study.

Trace elements solution	g/L
KBr	8
SrCl2x 6 H_2O	3.4
H_3BO_3	2.2
Na_2O_3Si	0.7
NaF	0.24
NH_4NO_3	0.16

Table S3 Results for ANOSIM and Adonis calculations comparing unweighted and weighted Unifrac matrix for 32 DNA and 32 RNA investigated samples obtained from the four *C. pagurus* specimens investigated in this study.

**Samples**	**Statistic**	**N**	**ANOSIM(R)**	**Adonis(R^2)**
DNA	Unweighted Unifrac	32	0.185	0.081
	Weighted Unifrac	32	0.178	0.118
RNA	Unweighted Unifrac	32	0.165	0.085
	Weighted Unifrac	32	0.155	0.117

Table S4 Similarity of the 16S rRNA genes of isolated strains in comparison to OTUs of the V3-V4 amplicon sequences, sorted into four categories according to their percentages of correlations: = 100%, ≥ 99%, ≥ 98% and ≥ 97%. Percentages of the genetic divergences between isolates and corresponding OTUs are shown in parenthesis. OTU consensus sequences representing more than 1% of the total epibacterial community on the carapace of *C. pagurus* are marked in blue (found in NA areas) and red (found in BS areas).

Similarity	Isolate		Corresponding OTU (genetic divergence %)							
100 %	CP8	*Aquimarina* sp.	OTU_2019	(0.0)						
	CP32	*Paracoccus* sp.	OTU_46	(0.0)						
	CP58	*Sphingorhabdus* sp.	OTU_200	(0.0)						
	CP68	*Vibrio* sp.	OTU_646	(0.0)						
	CP70	*Psychrobacillus* sp.	OTU_224	(0.0)						
	CP110	*Loktanella* sp.	OTU_2471	(0.0)	OTU_2557	(0.0)				
	CP113	*Aquimarina* sp.	OTU_2020	(0.0)						
	CP119	*Colwellia* sp.	OTU_86	(0.0)						
	CP123	*Leucothrix* sp.	OTU_123	(0.0)						
	CP148	*Sulfitobacter* sp.	**OTU_17**	(0.0)						
	CP157	*Paracoccus* sp.	OTU_2557	(0.0)						
	CP193	*Vibrio* sp.	OTU_491	(0.0)						
99 %	CP62	*Vibrio* sp.	OTU_491	(0.9)						
	CP97	*Aerococcus* sp.	OTU_2382	(0.7)						
	CP110	*Loktanella* sp.	**OTU_2**	(0.5)						
	CP122	*Leucothrix* sp.	OTU_906	(0.2)						
	CP132	*Roseovarius* sp.	OTU_2688	(0.5)						
	CP157	*Paracoccus* sp.	**OTU_2**	(0.5)						
98 %	CP11	*Aquimarina* sp.	**OTU_1**	(1.4)						
	CP22	*Aquimarina* sp.	**OTU_1**	(1.4)						
	CP23	*Aquimarina* sp.	**OTU_1**	(1.4)						
	CP57	*Maribacter* sp.	OTU_2499	(1.1)						
	CP113	*Aquimarina* sp.	OTU_2518	(1.6)						
	CP123	*Leucothrix* sp.	OTU_906	(1.9)						
97 %	CP1	*Lacinutrix* sp.	OTU_574	(2.4)						
	CP2	*Bacillus* sp.	OTU_1956	(2.8)	OTU_2123	(2.8)	OTU_2123	(2.7)		
	CP5	*Bacillus* sp.	OTU_2123	(2.5)						
	CP8	*Aquimarina* sp.	**OTU_32**	(2.8)						
	CP14	*Tenacibaculum* sp.	OTU_2765	(2.4)						
	CP16	*Sphingomonas* sp.	OTU_2982	(2.8)						
	CP32	*Paracoccus* sp.	OTU_2982	(2.8)						
	CP41	*Vibrio* sp.	OTU_1124	(2.7)						
	CP52	*Tenacibaculum* sp.	OTU_2765	(2.8)						
	CP58	*Sphingorhabdus* sp.	OTU_1944	(2.5)						
	CP74	*Colwellia* sp.	OTU_2040	(2.8)						
	CP103	*Pseudoalteromonas* sp.	OTU_537	(2.8)						
	CP105	*Psychrobacter* sp.	OTU_2605	(2.7)						
	CP110	*Loktanella* sp.	OTU_346	(2.6)						
	CP112	*Bacillus* sp.	OTU_814	(2.6)						
	CP113	*Aquimarina* sp.	**OTU_32**	(2.7)	OTU_2231	(2.8)				
	CP118	*Winogradskyella* sp.	OTU_428	(2.7)	OTU_1277	(2.7)	OTU_1836			
	CP119	*Colwellia* sp.	OTU_2506	(2.7)	OTU_931	(2.4)				
	CP122	*Leucothrix* sp.	OTU_2815	(2.4)						
	CP123	*Leucothrix* sp.	OTU_2815	(2.7)						
	CP124	*Pseudoalteromonas* sp.	OTU_537	(2.8)						
	CP132	*Roseovarius* sp.	OTU_2552	(2.4)						
	CP135	*Octadecabacter* sp.	OTU_384	(2.8)						
	CP141	*Staphylococcus* sp.	OTU_1354	(2.7)	OTU_565	(2.9)	OTU_835	(2.8)		
	CP157	*Paracoccus* sp.	OTU_346	(2.4)						
	CP190	*Cocleimonas* sp.	OTU_1919	(2.5)	**OTU_13**	(2.7)				
	CP194	*Maribacter* sp.	OTU_2518	(2.8)	OTU_2231	(2.4)	OTU_2499	(2.7)		
	CP200	*Cocleimonas* sp.	OTU_1919	(2.7)	OTU_1962	(2.8)	OTU_2232	(2.8)	**OTU_13**	(2.9)

> 1 % of the community in non-affected areas

> 1 % of the community in black spot affected areas

22 **Table S5** Positive results obtained with the agar diffusion test, screening for antagonistic activities of isolates obtained from *Cancer pagurus* (first column), tested against standard laboratory strains and against strains also isolated from *C. pagurus* (isolates with prefix CP). Grey background showed strains isolated from black-spot affected areas. Zones of inhibition are defined by the following symbols: + >2 mm; ++ >4 mm; +++ >6 mm.

CP360 *Bacillus* sp.	CP157 *Paracoccus* sp.	CP141 *Staphylococcus* sp.	CP139 *Vibrio* sp.	CP134 *Vibrio* sp.	CP124 *Pseudoalteromonas* sp.	CP123 *Leucothrix* sp.	CP112 *Bacillus* sp.	CP111 *Aquimarina* sp.	CP103 *Pseudoalteromonas* sp.	CP78 *Bacillus* sp.	CP68 *Vibrio* sp.	CP62 *Vibrio* sp.	CP52 *Tenacibaculum* sp.	CP51 *Aquimarina* sp.	CP50 *Vibrio* sp.	CP41 *Vibrio* sp.	CP29 *Vibrio* sp.	CP22 *Aquimarina* sp.	CP19 *Aquimarina* sp.	CP14 *Tenacibaculum* sp.	CP11 *Aquimarina* sp.	CP8 *Aquimarina* sp.	CP5 *Bacillus* sp.	Strain		
			+	+				++	+		++		+			+			+	+		+		1786	*Loktanella pyoseonensis*	*α-Proteobacteria*
		+											+							++			+	1810	*Pantoea* sp.	
++													+						+	+				WHV03	*Vibrio corallilyticus*	*γ-Proteobacteria*
			+	+					+		+	+	+				+			+				1621	*Aliivibrio fischeri*	
																								1632	*Aquimarina intermedia*	*Flavobacteria*
													+				+			++				1682	*Rhodococcus corynebacterioides*	
+			+		+									+	+					++				1701	*Kocuria sp.*	*Actinobacteria*
++		+++			++	++	+				+++		+	+	+			+		+	+	+	+	1718	*Staphylococcus sp .*	*Bacilli*
+					++	++				++	++			++										1719	*Exiguobacterium sp.*	
																								CP110	*Loktanella* sp.	*α-Proteobacteria*
+										+	+++			+			+			+	+	++		CP 35	*Paracoccus* sp.	
													+							+				CP98	*Vibrio* sp.	*γ-Proteobacteria*
																								CP193	*Vibrio* sp.	
											+++									+				CP123	*Leucothrix* sp.	
+++													+							+				CP23	*Shewanella* sp.	
											+		+							++				CP190	*Cocleiomonas* sp.	
+									+					+							+	+		CP30	*Arthrobacter* sp.	*Actinobacteria*
											+++													CP102	*Microbacterium* sp.	
+											+++		+											CP112	*Brevibacterium* sp.	
+			+								+		+							++				CP97	*Aerococcus* sp.	*Bacilli*
													+							+				CP1	*Nonlabens* sp.	*Flavobacteriia*
																		+		+				CP8	*Aquimarina* sp	
	++		+											++							+	++		CP14	*Tenacibaculum* sp.	
											+++									+				CP16	*Sphingomonas* sp.	
											+++		+							++				CP161	*Lutibacter* sp.	
											+++		+							+				CP191	*Lutimonas* sp.	

27

Fig. S1. Rarefaction curves based on V3-V4 amplicon sequencing data obtained for (a) individual samples (in total 64 samples) and (b) for samples from the four specimens of *Cancer pagurus*. For details concerning sample preparation and treatment see Text S1.

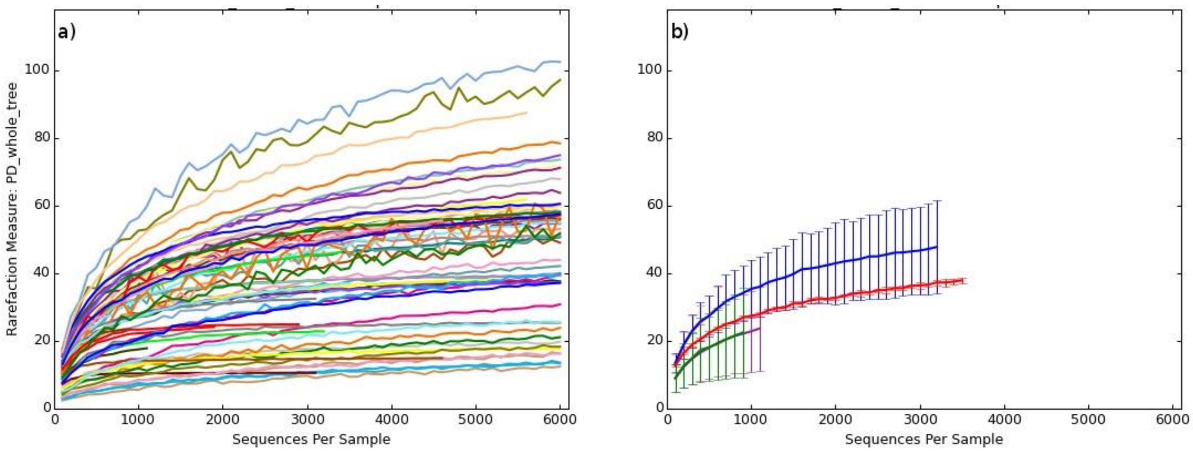

Text S1

MiSeq library preparations. LGC Genomics (Berlin, Germany) prepared and sequenced barcode amplicon libraries of the hypervariable regions V3 to V4 of the 16S rRNA gene fragments, comprising all 67 samples analysed in this study. The V3-V4 region was amplified using the barcode primers 341F (5´-CCTACGGGNGGCWGCAG-3´) and 785R (5´-GACTACHVGGGTATCTAAKCC-3´)[1]. Barcodes were unique for each sample. PCRs were performed by LGC using 1 µl diluted template (1:50), 15 pmol of each barcode labeled primer, 1.5 units of MyTaq™ DNA polymerase (Bioline, London UK) and 2 µl BIoStab PCR Optimizer II (Sigma-Aldrich, St. Louis, Missouri, USA) in 20 µl MyTaq buffer. PCR comprised 25 cycles of 96°C for 15 sec, 50 °C for 30 sec, and 70°C for 60 sec. Concentrations of amplicons were determined by using gel electrophoresis. About 20 ng of each amplicon were pooled into 48 sample amplicon pools. Amplicon pools were purified using AMPure beads (Beckman-Coulter, Krefeld, Germany) and MinElute columns (Qiagen, Hilden, Germany) to remove primer dimers. Afterwards LGC constructed Illumina libraries using the Ovation Rapid DR Multiplex System 1-96 (NuGEN Technologies, Inc., San Carlos, CA, USA) followed by MiSeq sequencing (Illumina, San Diego, California, USA). LGC genomics sorted fasta files by barcodes, removed adapters and barcode sequences and rejected sequences with missing or incompatible barcodes using BCL2 Fastq Version 1.8.4 (Illumina, San Diego, California, USA).

Demultiplexing and statistical analysis. A total of 3,203,275 paired end reads was sequenced by Illumina Miseq V3-V4, derived from 67 samples. Average sequencing depth was 33,405 +- 27,165 reads. After merging paired end reads with a first quality control 1,768,840 combined reads were further processed. A second filter step removed 38,792 reads that 1,730,048 reads (698,846 are end-trimmed) remained for OTU clustering with an average length of 336. After dereplication 61,133 sequences with sufficient abundance were clustered

into 2989 OTUs. Blast analysis resulted in a classification of 99.9% of the OTUs on phylum level, 88.1% at family level, 65.4% on genus level and 15.9% on species level. We selectively excluded taxa that were of insufficient abundance (<1%) to be statistically significant.

Rarefaction analysis indicated that all 67 samples were close to saturation (see Fig. S2 in the supplemental material). The increase of phylogenetic diversity with sampled reads per sample already flattens for many samples at ~1000 reads per pool. For few samples phylogenetic diversity even remained constant after this point. Some samples showed a slight increase of phylogenetic diversity with increasing sequencing effort even after 2000 reads sequenced per pool. For all further analyses we rarefied the communities to 2000 reads per pool. The different groups of samples showed a different increase of phylogenetic diversity with read abundance.

From a total of 67 samples 2.5 million 300 bp paired end reads were sequenced in two separate libraries using the Illumina MiSeq V3 platform. Raw reads were demultiplexed using bcl2fastq 1.8.4 software (Illumina) allowing for a maximum of two mismatches in barcodes and three mismatches in primer sequence. Primer and barcodes were clipped from each read. Paired reads were combined using BBMerge 34.48 (https://sourceforge.net/projects/bbmap/). Demultiplexed and combined reads for each of the 67 samples were then analyzed using LOTUs pipeline version 1.512 including quality filtering by sdm 1.27 beta[2]. Reads were first trimmed for average quality in 15 bp windows to be higher than 20 and trimmed when two errors in sequence were accumulated. Reads were discarded after trimming when outside of the range 100-500 bp, lower than 25 average quality or more than two ambiguous bases were found in the sequence. High quality reads were clustered *de novo* by UPARSE algorithm[3,4], with 99 % similarity. OTUs were checked for chimeras using VSEARCH 1.13 algorithm[5] against RDP Gold reference database (http://drive5.com/uchime/gold.fa). Each OTU was assigned by blast algorithm[6] against the SILVA 16S rDNA database[7,8]. Taxonomic classification was performed from phylum to species level. For phylogenetic inference for OTUs a multiple sequence alignment was calculated by Clustal-omega[9] and used to construct the phylogenetic tree by

FastTree2 using the default gamma model of the LOTUs pipeline[2]. The raw files provided for each sample and the OTUs calculated based on 99% similarity among reads were uploaded to the European Nucleotide Archive and can be found under the accession number PRJEB40004.

Statistical analysis for the calculation of the diversity was performed with Qiime 1.9.0[10]. Alpha diversity was calculated based on Chao and phylogenetic diversity. Differences of alpha diversity between treatments were analyzed using R version 3.2.3[11], based on Shannon-Wiener index and Simpson evenness, calculated as inverse Simpson index divided by species richness. Reads in each sampling pool were rarefied to 3000 reads to correct for sampling depth heterogeneity. Beta diversity was calculated using Bray-Curtis dissimilarity as well as weighted and unweighted UniFrac dissimilarity[12]. Confidence intervals were calculated based on jackknifing to 2000 reads per sample. The vegan package in R[13] was used to perform the SIMPER analysis in order to calculate the contribution of different OTUs for the similarity of samples among groups. Different plots included in the Qiime 1.9.0 pipeline[10], such as Principle Coordinate Analysis (PCoA), were used to visualize diversity among samples. All further statistics were calculated separately for the pools of samples from the four different treatments: Black Spot DNA (BS_DNA), Non-Affected DNA (NA_DNA), Black Spot RNA (BS_RNA) and Non-Affected RNA (NA_RNA). The core community of each treatment was defined as the OTUs which are present in 90% of the samples for each group. OTU frequencies between samples from BS and NA for the core communities were compared by Mann Whitney U test including bonferroni correction for multiple comparisons using Qiime 1.9.0[10].

References

1. Herlemann, D. P., Labrenz, M., Jürgens, K., Bertilsson, S., Waniek, J. J., & Andersson, A. F. (2011). Transitions in bacterial communities along the 2000 km salinity gradient of the Baltic Sea. The ISME journal, 5(10), 1571.

2. Hildebrand, F., Tadeo, R., Voigt, A. Y., Bork, P., & Raes, J. (2014). LotuS: an efficient and user-friendly OTU processing pipeline. Microbiome, 2(1), 30.

3. Edgar, R. C. (2013). UPARSE: highly accurate OTU sequences from microbial amplicon reads. Nature methods, 10(10), 996.

4. Edgar, R. C., Haas, B. J., Clemente, J. C., Quince, C., & Knight, R. (2011). UCHIME improves sensitivity and speed of chimera detection. Bioinformatics, 27(16), 2194-2200.

5. Rognes T (2015) VSEARCH 1.13 (chimera de novo / ref; OTU alignments) https://github.com/torognes/vsearch

6. Altschul, S. F., Gish, W., Miller, W., Myers, E. W., & Lipman, D. J. (1990). Basic local alignment search tool. Journal of molecular biology, 215(3), 403-410.

7. SILVA 16S/18S database - Yilmaz P, Parfrey LW, Yarza P, Gerken J, Pruesse E, Quast C, Schweer T, Peplies J, Ludwig W, Glockner FO (2014) The SILVA and "All-species Living Tree Project (LTP)" taxonomic frameworks. Nucleic Acid Res. 42:D643-D648

8. Quast, C., Pruesse, E., Yilmaz, P., Gerken, J., Schweer, T., Yarza, P., ... & Glöckner, F. O. (2012). The SILVA ribosomal RNA gene database project: improved data processing and web-based tools. Nucleic acids research, gks1219.

9. Sievers, F., Wilm, A., Dineen, D., Gibson, T. J., Karplus, K., Li, W., ... & Thompson, J. D. (2011). Fast, scalable generation of high-quality protein multiple sequence alignments using Clustal Omega. Molecular systems biology, 7(1), 539.

10. Caporaso, J. G., Kuczynski, J., Stombaugh, J., Bittinger, K., Bushman, F. D., Costello, E. K., Huttley, G. A. (2010). QIIME allows analysis of high-throughput community sequencing data. Nature methods, 7(5), 335.

11. Team, R. C. (2015). R: A language and environment for statistical computing.

12. Lozupone, C., & Knight, R. (2005). UniFrac: a new phylogenetic method for comparing microbial communities. Applied and environmental microbiology, 71(12), 8228-8235.

13. Oksanen, J., Blanchet, F. G., Kindt, R., Legendre, P., Minchin, P. R., O'hara, R. B., ... & Wagner, H. (2011). vegan: Community ecology package. R package version, 117-118.
